# Supplementary figures and images for: Apobec-mediated retroviral hypermutation in vivo is dependent on mouse strain
Source: PLoS Pathog. 2024 Aug 29;20(8):e1012505. doi: 10.1371/journal.ppat.1012505 (PMC11389910; doi:10.1371/journal.ppat.1012505)

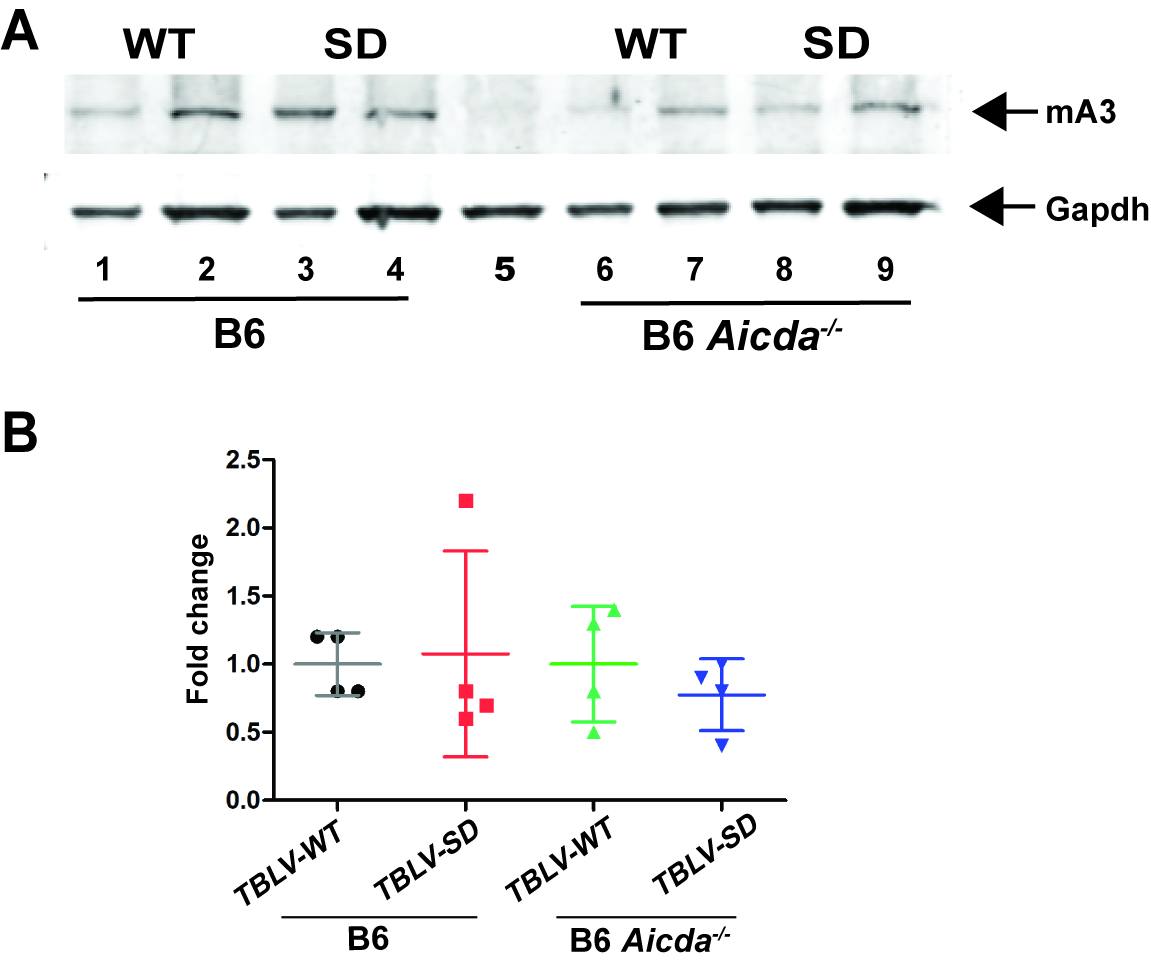

Supplement: S1 Fig — (A) Representative Western blot analysis for mA3 levels in individual tumors. Total protein was extracted from individual T-cell tumors induced by TBLV-WT or TBLV-SD and subjected to Western blotting with antibodies specific for mA3 or Gapdh. Four tumor-derived protein lysates from each virus/mouse strain combination were used for analysis. Results are shown for two B6 tumors induced by TBLV-WT (lanes 1 and 2), two B6 tumors induced by TBLV-SD (lanes 3 and 4), one B6 mA3/Aicda-double knockout tumor induced by TBLV-WT (lane 5), two B6 Aicda-/- tumors induced by TBLV-WT (lanes 6 and 7), and two B6 Aicda-/- tumors induced by TBLV-SD (lanes 8 and 9). A faint background band is observed in tumor extracts from mice lacking mA3 expression (lane 5). (B) Quantitation of mA3 levels in TBLV-induced T-cell tumors. The mA3 levels in each of four B6 tumors induced by TBLV-WT (black dots) were quantitated by LI-COR software relative to Gapdh, and the mean was assigned a relative value of 1.0 (horizontal line). The vertical bars show the standard deviations from the means. The mA3 levels of four independent TBLV-SD-induced B6 tumors (red squares), TBLV-WT-induced B6 Aicda-/- tumors (green triangles), and TBLV-SD-induced B6 Aicda-/- tumors (blue inverted triangles) are shown. Means for each group (horizontal lines) relative to the mean of values from four TBLV-WT-induced B6 tumors are shown by horizontal lines. Standard deviations from the means are shown by vertical bars. No statistical differences were observed. (TIF) [file ppat.1012505.s001.tif]
