# Supplementary material for: Apobec-mediated retroviral hypermutation in vivo is dependent on mouse strain
Source: PLoS Pathog. 2024 Aug 29;20(8):e1012505. doi: 10.1371/journal.ppat.1012505 (PMC11389910; doi:10.1371/journal.ppat.1012505)
Supplement: S1 Table — (PDF) [file ppat.1012505.s002.pdf]

gene_nam	padj	log2fc	sigGene	CatShort	gene_type	description
Ighg3	0.052973	-2.52803	gl.sig_4b_d	Innate	IG_C_gene	Immunoglobulin heavy constant g
C4b	0.097307	-2.55125	gl.sig_4b_d	Innate	protein_co	complement component 4B (Chido
Fgfr2	0.007458	4.686938	not_gl.sig_1a_up		protein_co	fibroblast growth factor receptor 2
Gm11878	0.007458	21.27792	not_gl.sig_1a_up		processed_	predicted gene 11878
Mpzl2	0.007586	3.819387	not_gl.sig_1a_up		protein_co	myelin protein zero-like 2
Sh3pxd2a	0.009007	1.837458	not_gl.sig_2a_up		protein_co	SH3 and PX domains 2A
Prdm11	0.040868	2.56965	not_gl.sig_2a_up		protein_co	PR domain containing 11
Dmxl1	0.02942	-1.71043	not_gl.sig_2b_dn		protein_co	Dmx-like 1
Fchsd2	0.025067	0.934905	not_gl.sig_3a_up		protein_co	FCH and double SH3 domains 2
Setd7	0.040868	-0.74229	not_gl.sig_3b_dn		protein_co	SET domain containing (lysine met
Msmo1	0.051428	1.194093	not_gl.sig_4a_up		protein_co	methylsterol monoxygenase 1
Angptl2	0.052973	1.567783	not_gl.sig_4a_up		protein_co	angiopoietin-like 2
Lmcd1	0.052973	4.544548	not_gl.sig_4a_up		protein_co	LIM and cysteine-rich domains 1
Efnb2	0.06636	3.477411	not_gl.sig_4a_up		protein_co	ephrin B2
Dlgap1	0.06636	4.033903	not_gl.sig_4a_up		protein_co	DLG associated protein 1
Ppef2	0.06636	3.523659	not_gl.sig_4a_up		protein_co	protein phosphatase, EF hand calc
Ankmy1	0.06636	1.883185	not_gl.sig_4a_up		protein_co	ankyrin repeat and MYND domain
Cd160	0.06636	2.498164	not_gl.sig_4a_up		protein_co	CD160 antigen
Klc3	0.06636	2.380266	not_gl.sig_4a_up		protein_co	kinesin light chain 3
Zbtb10	0.06636	5.550844	not_gl.sig_4a_up		protein_co	zinc finger and BTB domain contai
Ugt3a1	0.06636	3.457244	not_gl.sig_4a_up		protein_co	UDP glycosyltransferases 3 family,
Vmn1r236	0.074886	2.614574	not_gl.sig_4a_up		protein_co	vomeronasal 1 receptor 236
Gm30400	0.074886	2.996896	not_gl.sig_4a_up		lncRNA	predicted gene, 30400
Fbln1	0.097307	1.177254	not_gl.sig_4a_up		protein_co	fibulin 1
Stc1	0.097307	3.248842	not_gl.sig_4a_up		protein_co	stanniocalcin 1
Trib3	0.097307	1.977332	not_gl.sig_4a_up		protein_co	tribbles pseudokinase 3
Lss	0.097307	1.116961	not_gl.sig_4a_up		protein_co	lanosterol synthase
Prr36	0.097307	5.855212	not_gl.sig_4a_up		protein_co	proline rich 36
Acot1	0.097307	2.177445	not_gl.sig_4a_up		protein_co	acyl-CoA thioesterase 1
Mir100hg	0.097307	3.082289	not_gl.sig_4a_up		lncRNA	Mir100 Mirlet7a-2 Mir125b-1 clus
Zfp111	0.097307	2.492884	not_gl.sig_4a_up		protein_co	zinc finger protein 111
Gm37102	0.097307	1.628646	not_gl.sig_4a_up		processed_	predicted gene, 37102
Gm45105	0.097307	2.860109	not_gl.sig_4a_up		processed_	predicted gene 45105
Gm47933	0.097307	2.003212	not_gl.sig_4a_up		processed_	predicted gene, 47933
Gm3219	0.097307	0.915142	not_gl.sig_4a_up		processed_	predicted pseudogene 3219
Gm49534	0.097307	1.835917	not_gl.sig_4a_up		lncRNA	predicted gene, 49534
Comt	0.06636	-1.28369	not_gl.sig_4b_dn		protein_co	catechol-O-methyltransferase
Hps1	0.06636	-1.01146	not_gl.sig_4b_dn		protein_co	HPS1, biogenesis of lysosomal orga
Tubb2b	0.06636	-2.13693	not_gl.sig_4b_dn		protein_co	tubulin, beta 2B class IIB
Bola3	0.06636	-0.56419	not_gl.sig_4b_dn		protein_co	bolA-like 3 (E. coli)
Iglc3	0.06636	-4.90421	not_gl.sig_4b_dn		IG_C_gene	immunoglobulin lambda constant
Fkbp2	0.06649	-0.91675	not_gl.sig_4b_dn		protein_co	FK506 binding protein 2
Tubb2a	0.077313	-1.78006	not_gl.sig_4b_dn		protein_co	tubulin, beta 2A class IIA
Kmo	0.097307	-2.37197	not_gl.sig_4b_dn		protein_co	kynurenine 3-monooxygenase (kyı
H2aj	0.097307	-0.77272	not_gl.sig_4b_dn		protein_co	H2J.A histone
Serf2	0.007586	-0.49878	not_gl.sig_5b_dn		protein_co	small EDRK-rich factor 2

anelles complex 3 subunit 1

nurenine 3-hydroxylase)
