# Supplementary material for: Apobec-mediated retroviral hypermutation in vivo is dependent on mouse strain
Source: PLoS Pathog. 2024 Aug 29;20(8):e1012505. doi: 10.1371/journal.ppat.1012505 (PMC11389910; doi:10.1371/journal.ppat.1012505)
Supplement: S2 Table — (PDF) [file ppat.1012505.s003.pdf]

gene_name	padj	log2fc	sigGene	CatShort	gene_type	description
Socs3	1.80E-05	2.926414	gl.sig_1a_u	Cytok,IFNs	protein_co	suppressor of cytokine signaling 3
Gpr183	0.00031	2.811816	gl.sig_1a_u	Inflam	protein_co	G protein-coupled receptor 183
Cd82	0.001494	2.108889	gl.sig_1a_u	Inflam	protein_co	CD82 antigen
Inhba	0.001494	4.719047	gl.sig_1a_u	Inflam	protein_co	inhibin beta-A
Ifi211	0.001565	2.992804	gl.sig_1a_u	Innate	protein_co	interferon activated gene 211
Ifi204	0.001624	2.491901	gl.sig_1a_u	Innate	protein_co	interferon activated gene 204
Dusp6	0.003084	2.286859	gl.sig_1a_u	Cytok,Innat	protein_co	dual specificity phosphatase 6
Atp8b4	0.003639	2.956558	gl.sig_1a_u	Innate	protein_co	ATPase, class I, type 8B, member 4
Ifitm2	0.007566	1.805489	gl.sig_2a_u	IFNs	protein_co	interferon induced transmembran
Trem2	0.008313	1.781446	gl.sig_2a_u	Innate	protein_co	triggering receptor expressed on n
Cpne3	0.010368	1.701667	gl.sig_2a_u	Innate	protein_co	copine III
Il6ra	0.011476	1.673451	gl.sig_2a_u	Cytok	protein_co	interleukin 6 receptor, alpha
Il2ra	0.012871	4.409025	gl.sig_2a_u	Cytok,Inflar	protein_co	interleukin 2 receptor, alpha chain
Plac8	0.016208	2.452046	gl.sig_2a_u	Innate	protein_co	placenta-specific 8
Stat1	0.016228	1.174103	gl.sig_2a_u	IFNs	protein_co	signal transducer and activator of
Ahr	0.017925	2.693176	gl.sig_2a_u	Inflam	protein_co	aryl-hydrocarbon receptor
Stat4	0.021652	3.022195	gl.sig_2a_u	IFNs	protein_co	signal transducer and activator of
Tnfaip2	0.026181	1.448298	gl.sig_2a_u	IFNs	protein_co	tumor necrosis factor, alpha-induc
Ptprj	0.02837	1.26915	gl.sig_2a_u	Cytok,Innat	protein_co	protein tyrosine phosphatase, rece
Lamp2	0.028913	1.056259	gl.sig_2a_u	Innate	protein_co	lysosomal-associated membrane p
P2ry14	0.029923	1.476783	gl.sig_2a_u	IFNs	protein_co	purinergic receptor P2Y, G-protein
Dnm3	0.03028	2.205376	gl.sig_2a_u	Innate,TLRs	protein_co	dynamin 3
Fcgr1	0.035434	1.8672	gl.sig_2a_u	IFNs,Innate	protein_co	Fc receptor, IgG, high affinity I
Rab31	0.03672	1.968762	gl.sig_2a_u	Innate	protein_co	RAB31, member RAS oncogene far
S100a11	0.03791	1.107721	gl.sig_2a_u	Innate	protein_co	S100 calcium binding protein A11
Col1a2	0.039327	1.892624	gl.sig_2a_u	Inflam	protein_co	collagen, type I, alpha 2
Lgmn	0.044029	1.346233	gl.sig_2a_u	Innate,TLRs	protein_co	legumain
Tent5a	0.045235	1.450836	gl.sig_2a_u	IFNs	protein_co	terminal nucleotidyltransferase 5A
Col1a1	0.045272	1.883705	gl.sig_2a_u	Inflam	protein_co	collagen, type I, alpha 1
Fyn	0.045502	1.06202	gl.sig_2a_u	Cytok,Innat	protein_co	Fyn proto-oncogene
Cd44	0.047955	1.914357	gl.sig_2a_u	Cells,Innate	protein_co	CD44 antigen
Diaph1	9.94E-05	0.847528	gl.sig_3a_u	Innate	protein_co	diaphanous related formin 1
Ptpre	0.002639	0.643137	gl.sig_3a_u	Inflam	protein_co	protein tyrosine phosphatase, rece
Rnf213	0.02634	0.87201	gl.sig_3a_u	IFNs	protein_co	ring finger protein 213
Tnfrsf1b	0.028913	0.92578	gl.sig_3a_u	Cytok,Inflar	protein_co	tumor necrosis factor receptor sup
Pim1	0.02922	0.962204	gl.sig_3a_u	IFNs	protein_co	proviral integration site 1
Pml	0.034576	0.588649	gl.sig_3a_u	IFNs	protein_co	promyelocytic leukemia
Plcg1	0.037604	0.752142	gl.sig_3a_u	AntiV,Cytol	protein_co	phospholipase C, gamma 1
Gm2a	0.037759	0.960514	gl.sig_3a_u	Innate	protein_co	GM2 ganglioside activator protein
Panx1	0.038627	0.774295	gl.sig_3a_u	Inflam,Inna	protein_co	pannexin 1
Tyk2	0.041837	0.716748	gl.sig_3a_u	Cytok,IFNs	protein_co	tyrosine kinase 2
Galns	0.04373	0.933513	gl.sig_3a_u	Innate	protein_co	galactosamine (N-acetyl)-6-sulfate
Skp1	0.007669	-0.74061	gl.sig_3b_d	Cytok,Innat	protein_co	S-phase kinase-associated protein
Eif4a1	0.009019	-0.62353	gl.sig_3b_d	AntiV,Cytol	protein_co	eukaryotic translation initiation fa
Psma6	0.016841	-0.69708	gl.sig_3b_d	Cytok,Innat	protein_co	proteasome subunit alpha 6
Nfatc3	0.041784	-0.72005	gl.sig_3b_d	Innate	protein_co	nuclear factor of activated T cells,

Nup93	0.043796	-0.56921	gl.sig_3b_d	IFNs	protein_co	nucleoporin 93
Cd93	0.050342	1.724827	gl.sig_4a_u	Innate	protein_co	CD93 antigen
Cd274	0.054394	1.118175	gl.sig_4a_u	IFNs	protein_co	CD274 antigen
Lcn2	0.057501	2.209616	gl.sig_4a_u	Innate	protein_co	lipocalin 2
C3ar1	0.058327	1.372324	gl.sig_4a_u	Inflam,Inna	protein_co	complement component 3a recep
Ncf1	0.058714	1.645372	gl.sig_4a_u	Innate	protein_co	neutrophil cytosolic factor 1
F2	0.060888	3.488478	gl.sig_4a_u	Innate	protein_co	coagulation factor II
Rab3d	0.061229	1.386342	gl.sig_4a_u	Innate	protein_co	RAB3D, member RAS oncogene fa
Clec12a	0.062147	1.494781	gl.sig_4a_u	Innate	protein_co	C-type lectin domain family 12, me
Serpinb6a	0.062147	0.832442	gl.sig_4a_u	Innate	protein_co	serine (or cysteine) peptidase inhil
Csf2rb	0.062748	1.299114	gl.sig_4a_u	Cytok	protein_co	colony stimulating factor 2 recepto
Lifr	0.064415	1.510854	gl.sig_4a_u	Cytok	protein_co	LIF receptor alpha
Rnf31	0.064415	0.680058	gl.sig_4a_u	IFNs	protein_co	ring finger protein 31
Slc11a1	0.064524	1.896047	gl.sig_4a_u	Innate	protein_co	solute carrier family 11 (proton-co
Cd36	0.064524	1.631081	gl.sig_4a_u	Innate,TLRs	protein_co	CD36 molecule
Il1rn	0.065847	2.558366	gl.sig_4a_u	Cytok	protein_co	interleukin 1 receptor antagonist
Znfx1	0.069158	0.899123	gl.sig_4a_u	IFNs	protein_co	zinc finger, NFX1-type containing 1
Il15ra	0.070354	1.864456	gl.sig_4a_u	Cytok,IFNs,	protein_co	interleukin 15 receptor, alpha chai
Tmc6	0.072538	0.57685	gl.sig_4a_u	Innate	protein_co	transmembrane channel-like gene
Pdgfa	0.073307	1.705801	gl.sig_4a_u	Cytok,Inflai	protein_co	platelet derived growth factor, alp
Stab1	0.073683	1.36987	gl.sig_4a_u	Inflam	protein_co	stabilin 1
Ikbke	0.083737	0.972637	gl.sig_4a_u	Innate,TLRs	protein_co	inhibitor of kappaB kinase epsilon
Mov10	0.084133	1.019059	gl.sig_4a_u	IFNs	protein_co	Mov10 RISC complex RNA helicase
Folr2	0.086786	2.074144	gl.sig_4a_u	Innate	protein_co	folate receptor 2 (fetal)
Mgst1	0.089015	1.61626	gl.sig_4a_u	Innate	protein_co	microsomal glutathione S-transfer
Msr1	0.089345	1.917784	gl.sig_4a_u	Inflam	protein_co	macrophage scavenger receptor 1
Fuca2	0.091847	0.74103	gl.sig_4a_u	Innate	protein_co	fucosidase, alpha-L- 2, plasma
Il34	0.096245	1.497323	gl.sig_4a_u	Cytok	protein_co	interleukin 34
Camk2g	0.096245	0.943807	gl.sig_4a_u	Cytok,IFNs	protein_co	calcium/calmodulin-dependent pr
Pnp	0.097051	0.69598	gl.sig_4a_u	IFNs,Innate	protein_co	purine-nucleoside phosphorylase
Irf5	0.098082	0.880548	gl.sig_4a_u	IFNs	protein_co	interferon regulatory factor 5
Cd53	0.098683	1.398209	gl.sig_4a_u	Innate	protein_co	CD53 antigen
Hmgb1	0.051921	-0.58986	gl.sig_4b_d	Cytok,Innat	protein_co	high mobility group box 1
Cd59b	0.07492	-1.67791	gl.sig_4b_d	Innate	protein_co	CD59b antigen
Map2k6	0.096245	-0.75584	gl.sig_4b_d	Cytok,Innat	protein_co	mitogen-activated protein kinase k
Tmem30a	0.054244	0.484325	gl.sig_5a_u	Innate	protein_co	transmembrane protein 30A
Atp6v1h	0.063337	0.496547	gl.sig_5a_u	Innate	protein_co	ATPase, H+ transporting, lysosoma
Rock1	0.073683	0.293446	gl.sig_5a_u	Innate	protein_co	Rho-associated coiled-coil contain
Psma7	0.000767	-0.47489	gl.sig_5b_d	Cytok,Innat	protein_co	proteasome subunit alpha 7
Psmc3	0.003341	-0.44458	gl.sig_5b_d	Cytok,Innat	protein_co	proteasome (prosome, macropain
Psmc1	0.00723	-0.49789	gl.sig_5b_d	Cytok,Innat	protein_co	protease (prosome, macropain) 26
Psmb5	0.017925	-0.46779	gl.sig_5b_d	Cytok,Innat	protein_co	proteasome (prosome, macropain
Psmb7	0.031439	-0.41787	gl.sig_5b_d	Cytok,Innat	protein_co	proteasome (prosome, macropain
Psmb2	0.037759	-0.39731	gl.sig_5b_d	Cytok,IFNs,	protein_co	proteasome (prosome, macropain
Calm3	0.066604	-0.30852	gl.sig_5b_d	Innate	protein_co	calmodulin 3
Adam19	1.08E-06	4.780474	not_gl.sig_1a_up		protein_co	a disintegrin and metallopeptidase
Hes1	1.08E-06	4.715971	not_gl.sig_1a_up		protein_co	hes family bHLH transcription facto

Hsd11b1	4.00E-06	2.412959	not_gl.sig_1a_up	protein_co	hydroxysteroid 11-beta dehydroge
Capn5	4.54E-05	6.231289	not_gl.sig_1a_up	protein_co	calpain 5
Wdr25	6.61E-05	4.336346	not_gl.sig_1a_up	protein_co	WD repeat domain 25
Erg	0.00012	4.987934	not_gl.sig_1a_up	protein_co	ETS transcription factor
Tspan9	0.0003	4.030887	not_gl.sig_1a_up	protein_co	tetraspanin 9
Gm266	0.00031	7.883449	not_gl.sig_1a_up	protein_co	predicted gene 266
Gimap1os	0.00031	3.638136	not_gl.sig_1a_up	lncRNA	GTPase, IMAP family member 1, o
Gm38118	0.00031	3.409793	not_gl.sig_1a_up	TEC	predicted gene, 38118
Pdgfrb	0.000381	3.500528	not_gl.sig_1a_up	protein_co	platelet derived growth factor rec
Afdn	0.000623	4.550292	not_gl.sig_1a_up	protein_co	afadin, adherens junction formatic
Gm48283	0.000675	2.565925	not_gl.sig_1a_up	lncRNA	predicted gene, 48283
Ace	0.000739	4.435418	not_gl.sig_1a_up	protein_co	angiotensin I converting enzyme (
Ptgfrn	0.000904	2.328441	not_gl.sig_1a_up	protein_co	prostaglandin F2 receptor negative
Tshz2	0.001071	2.630127	not_gl.sig_1a_up	protein_co	teashirt zinc finger family member
Arhgap26	0.001167	2.469607	not_gl.sig_1a_up	protein_co	Rho GTPase activating protein 26
Pomgnt2	0.001167	5.844817	not_gl.sig_1a_up	protein_co	protein O-linked mannose beta 1,4
Serpinb6b	0.00119	2.80053	not_gl.sig_1a_up	protein_co	serine (or cysteine) peptidase inhil
Cntnap2	0.001494	5.991925	not_gl.sig_1a_up	protein_co	contactin associated protein-like 2
Dennd3	0.001547	2.60899	not_gl.sig_1a_up	protein_co	DENN/MADD domain containing 3
2700054A1	0.002805	3.999381	not_gl.sig_1a_up	lncRNA	RIKEN cDNA 2700054A10 gene
Zfp760	0.002957	3.177939	not_gl.sig_1a_up	protein_co	zinc finger protein 760
Kit	0.003084	3.766576	not_gl.sig_1a_up	protein_co	KIT proto-oncogene receptor tyros
Emid1	0.003084	2.615111	not_gl.sig_1a_up	protein_co	EMI domain containing 1
Tedc1	0.003084	2.241673	not_gl.sig_1a_up	protein_co	tubulin epsilon and delta complex
Gpsm1	0.003154	2.49573	not_gl.sig_1a_up	protein_co	G-protein signalling modulator 1 (
Arhgap5	0.003154	2.666511	not_gl.sig_1a_up	protein_co	Rho GTPase activating protein 5
Gm29183	0.003154	5.505022	not_gl.sig_1a_up	lncRNA	predicted gene 29183
Gm12840	0.003179	4.297577	not_gl.sig_1a_up	lncRNA	predicted gene 12840
Map2	0.003341	4.232371	not_gl.sig_1a_up	protein_co	microtubule-associated protein 2
Itga9	0.003341	2.307093	not_gl.sig_1a_up	protein_co	integrin alpha 9
Uaca	0.003639	3.732485	not_gl.sig_1a_up	protein_co	uveal autoantigen with coiled-coil
Maf	0.003795	2.028272	not_gl.sig_1a_up	protein_co	avian musculoaponeurotic fibrosar
Smok4a	0.003881	5.991465	not_gl.sig_1a_up	transcribec	sperm motility kinase 4A
Slc23a4	0.003916	3.376903	not_gl.sig_1a_up	protein_co	solute carrier family 23 member 4
Atp8a2	0.004358	4.590542	not_gl.sig_1a_up	protein_co	ATPase, aminophospholipid transp
Gria3	0.00444	3.648206	not_gl.sig_1a_up	protein_co	glutamate receptor, ionotropic, AM
Olfr1033	0.00444	3.507064	not_gl.sig_1a_up	protein_co	olfactory receptor 1033
Hsd17b11	0.004493	2.289964	not_gl.sig_1a_up	protein_co	hydroxysteroid (17-beta) dehydrog
Sall3	0.005168	4.996921	not_gl.sig_1a_up	protein_co	spalt like transcription factor 3
Fbp1	0.005263	5.428877	not_gl.sig_1a_up	protein_co	fructose bisphosphatase 1
Frmd4a	0.005467	2.160871	not_gl.sig_1a_up	protein_co	FERM domain containing 4A
Acot1	0.005467	2.317019	not_gl.sig_1a_up	protein_co	acyl-CoA thioesterase 1
Bcl2l14	0.005629	2.927258	not_gl.sig_1a_up	protein_co	BCL2-like 14 (apoptosis facilitator)
Rapgef2	0.006285	2.497942	not_gl.sig_1a_up	protein_co	Rap guanine nucleotide exchange
Gm48942	0.006285	3.014158	not_gl.sig_1a_up	lncRNA	predicted gene, 48942
Arhgef10l	0.006318	3.859265	not_gl.sig_1a_up	protein_co	Rho guanine nucleotide exchange
F630028O1	0.007986	3.078921	not_gl.sig_1a_up	lncRNA	RIKEN cDNA F630028O10 gene

Slc5a9	0.008059	5.288321	not_gl.sig_1a_up	protein_co	solute carrier family 5 (sodium/glu
Cyp39a1	0.008476	6.151685	not_gl.sig_1a_up	protein_co	cytochrome P450, family 39, subfa
AC160336.	0.009019	3.146405	not_gl.sig_1a_up	lncRNA	novel transcript
Spp1	0.009819	3.082787	not_gl.sig_1a_up	protein_co	secreted phosphoprotein 1
Gm42420	0.009966	3.007478	not_gl.sig_1a_up	protein_co	predicted gene, 42420
Gm11878	1.16E-12	-33.9978	not_gl.sig_1b_dn	processed_	predicted gene 11878
Hes6	1.47E-05	-2.1383	not_gl.sig_1b_dn	protein_co	hairy and enhancer of split 6
Ift122	1.47E-05	-2.58817	not_gl.sig_1b_dn	protein_co	intraflagellar transport 122
Gm24616	0.001071	-2.122	not_gl.sig_1b_dn	snoRNA	predicted gene, 24616
Xrra1	0.009	-2.56313	not_gl.sig_1b_dn	protein_co	X-ray radiation resistance associat
Six4	0.009966	-3.38923	not_gl.sig_1b_dn	protein_co	sine oculis-related homeobox 4
Atp13a2	0.000132	1.257136	not_gl.sig_2a_up	protein_co	ATPase type 13A2
Coro7	0.000389	1.672609	not_gl.sig_2a_up	protein_co	coronin 7
Gna12	0.000464	1.415521	not_gl.sig_2a_up	protein_co	guanine nucleotide binding proteir
Tcf4	0.000568	1.69824	not_gl.sig_2a_up	protein_co	transcription factor 4
Gm12254	0.000609	1.475689	not_gl.sig_2a_up	processed_	predicted gene 12254
Dram2	0.000644	1.255071	not_gl.sig_2a_up	protein_co	DNA-damage regulated autophagy
Morrbid	0.001196	1.595545	not_gl.sig_2a_up	lncRNA	myeloid RNA regulator of BCL2L11
Gm27206	0.002236	1.292274	not_gl.sig_2a_up	lncRNA	predicted gene 27206
Slc12a7	0.003124	1.351595	not_gl.sig_2a_up	protein_co	solute carrier family 12, member 7
Arid5a	0.003154	1.521968	not_gl.sig_2a_up	protein_co	AT rich interactive domain 5A (MR
Snx10	0.003154	1.733298	not_gl.sig_2a_up	protein_co	sorting nexin 10
Gm38356	0.003435	1.543316	not_gl.sig_2a_up	processed_	predicted gene, 38356
Clec2i	0.003795	1.432854	not_gl.sig_2a_up	protein_co	C-type lectin domain family 2, mer
Dhx34	0.004358	1.624687	not_gl.sig_2a_up	protein_co	DEAH (Asp-Glu-Ala-His) box polype
Lrp1	0.004495	1.995189	not_gl.sig_2a_up	protein_co	low density lipoprotein receptor-r
Cblb	0.005196	1.726568	not_gl.sig_2a_up	protein_co	Casitas B-lineage lymphoma b
Hdac4	0.005412	1.655573	not_gl.sig_2a_up	protein_co	histone deacetylase 4
Agrn	0.006318	1.724352	not_gl.sig_2a_up	protein_co	agrin
F13a1	0.006495	1.949148	not_gl.sig_2a_up	protein_co	coagulation factor XIII, A1 subunit
Frmd4b	0.00714	1.951632	not_gl.sig_2a_up	protein_co	FERM domain containing 4B
Ahnak	0.00714	1.53144	not_gl.sig_2a_up	protein_co	AHNAK nucleoprotein (desmoyoki
Id2	0.008059	1.540981	not_gl.sig_2a_up	protein_co	inhibitor of DNA binding 2
Tmem229b	0.009984	1.668412	not_gl.sig_2a_up	protein_co	transmembrane protein 229B
Actn1	0.01011	1.636604	not_gl.sig_2a_up	protein_co	actinin, alpha 1
Dmwd	0.010368	1.537343	not_gl.sig_2a_up	protein_co	dystrophia myotonica-containing V
Heg1	0.010368	1.390873	not_gl.sig_2a_up	protein_co	heart development protein with E
Lox	0.010376	2.40467	not_gl.sig_2a_up	protein_co	lysyl oxidase
Slc45a3	0.010598	3.659599	not_gl.sig_2a_up	protein_co	solute carrier family 45, member 3
Sidt1	0.010674	2.906404	not_gl.sig_2a_up	protein_co	SID1 transmembrane family, mem
Laptm4b	0.010867	2.395275	not_gl.sig_2a_up	protein_co	lysosomal-associated protein trans
Cfap20dc	0.010911	4.784422	not_gl.sig_2a_up	protein_co	CFAP20 domain containing
Oaz1-ps	0.010911	1.393681	not_gl.sig_2a_up	processed_	ornithine decarboxylase antizyme
Tm6sf1	0.010931	1.689069	not_gl.sig_2a_up	protein_co	transmembrane 6 superfamily me
Rab34	0.011055	2.095749	not_gl.sig_2a_up	protein_co	RAB34, member RAS oncogene far
Gm45088	0.011055	1.417669	not_gl.sig_2a_up	TEC	predicted gene 45088
Gm20186	0.011076	2.314473	not_gl.sig_2a_up	lncRNA	predicted gene, 20186

Mpzl2	0.011217	2.839011	not_gl.sig_2a_up	protein_co	myelin protein zero-like 2
Tnk2	0.01143	1.86913	not_gl.sig_2a_up	protein_co	tyrosine kinase, non-receptor, 2
Trf	0.01143	1.56999	not_gl.sig_2a_up	protein_co	transferrin
Ankrd33b	0.011638	4.780704	not_gl.sig_2a_up	protein_co	ankyrin repeat domain 33B
Card10	0.011828	3.070242	not_gl.sig_2a_up	protein_co	caspase recruitment domain famil
Slc25a27	0.011832	3.363299	not_gl.sig_2a_up	protein_co	solute carrier family 25, member 2
Naip5	0.012009	3.146741	not_gl.sig_2a_up	protein_co	NLR family, apoptosis inhibitory pr
Tlr13	0.012189	2.548917	not_gl.sig_2a_up	protein_co	toll-like receptor 13
Susd4	0.012565	6.19688	not_gl.sig_2a_up	protein_co	sushi domain containing 4
Spsb1	0.012565	2.491797	not_gl.sig_2a_up	protein_co	splA/ryanodine receptor domain a
Mia2	0.012646	1.620736	not_gl.sig_2a_up	protein_co	MIA SH3 domain ER export factor
Rasa3	0.012756	1.693831	not_gl.sig_2a_up	protein_co	RAS p21 protein activator 3
Cish	0.012756	3.352223	not_gl.sig_2a_up	protein_co	cytokine inducible SH2-containing
Gatm	0.012871	1.779138	not_gl.sig_2a_up	protein_co	glycine amidinotransferase (L-argi
Apoc2	0.013195	3.28008	not_gl.sig_2a_up	protein_co	apolipoprotein C-II
Gm30159	0.013195	4.629673	not_gl.sig_2a_up	lncRNA	predicted gene, 30159
Pde7a	0.013337	2.536973	not_gl.sig_2a_up	protein_co	phosphodiesterase 7A
Vwce	0.01339	3.243073	not_gl.sig_2a_up	protein_co	von Willebrand factor C and EGF d
2010016I1	0.014781	2.376603	not_gl.sig_2a_up	lncRNA	RIKEN cDNA 2010016I18 gene
Slc9a9	0.01505	1.679951	not_gl.sig_2a_up	protein_co	solute carrier family 9 (sodium/hy
Stc1	0.015501	3.121591	not_gl.sig_2a_up	protein_co	stanniocalcin 1
Dgka	0.015888	2.091305	not_gl.sig_2a_up	protein_co	diacylglycerol kinase, alpha
Xkr6	0.016228	2.664492	not_gl.sig_2a_up	protein_co	X-linked Kx blood group related 6
Pkp4	0.016307	2.12084	not_gl.sig_2a_up	protein_co	plakophilin 4
Naxd	0.016562	1.501434	not_gl.sig_2a_up	protein_co	NAD(P)HX dehydratase
Notch3	0.016562	2.850396	not_gl.sig_2a_up	protein_co	notch 3
Sh3pxd2a	0.016562	1.315649	not_gl.sig_2a_up	protein_co	SH3 and PX domains 2A
Dynlt3	0.016841	1.309396	not_gl.sig_2a_up	protein_co	dynein light chain Tctex-type 3
Bace1	0.017247	1.324201	not_gl.sig_2a_up	protein_co	beta-site APP cleaving enzyme 1
Necab1	0.017403	4.417297	not_gl.sig_2a_up	protein_co	N-terminal EF-hand calcium bindin
Pqlc3	0.017403	1.088013	not_gl.sig_2a_up	protein_co	PQ loop repeat containing
Gm38244	0.017606	2.973104	not_gl.sig_2a_up	TEC	predicted gene, 38244
Sypl	0.017925	1.062502	not_gl.sig_2a_up	protein_co	synaptophysin-like protein
Maged1	0.017925	1.478124	not_gl.sig_2a_up	protein_co	MAGE family member D1
5830468F0	0.017925	2.043553	not_gl.sig_2a_up	lncRNA	RIKEN cDNA 5830468F06 gene
Alcam	0.018118	3.110319	not_gl.sig_2a_up	protein_co	activated leukocyte cell adhesion r
Prag1	0.018178	3.313737	not_gl.sig_2a_up	protein_co	PEAK1 related kinase activating ps
Gria2	0.018246	4.318689	not_gl.sig_2a_up	protein_co	glutamate receptor, ionotropic, AM
Mta3	0.018866	1.428396	not_gl.sig_2a_up	protein_co	metastasis associated 3
A530088E0	0.019241	1.677616	not_gl.sig_2a_up	lncRNA	RIKEN cDNA A530088E08 gene
Gm45551	0.019989	1.996333	not_gl.sig_2a_up	unprocesse	predicted gene 45551
Chil3	0.020173	3.952035	not_gl.sig_2a_up	protein_co	chitinase-like 3
Gbp9	0.020472	2.109585	not_gl.sig_2a_up	protein_co	guanylate-binding protein 9
Tbcel	0.021023	1.198065	not_gl.sig_2a_up	protein_co	tubulin folding cofactor E-like
4933439C1	0.021023	1.622718	not_gl.sig_2a_up	lncRNA	RIKEN cDNA 4933439C10 gene
Ccl9	0.021046	2.254094	not_gl.sig_2a_up	protein_co	chemokine (C-C motif) ligand 9
Pipox	0.021277	3.137117	not_gl.sig_2a_up	protein_co	pipecolic acid oxidase

Mfsd13a	0.021277	2.83771	not_gl.sig_2a_up	protein_co	major facilitator superfamily doma
Gm50287	0.021277	3.040822	not_gl.sig_2a_up	lncRNA	predicted gene, 50287
Jaml	0.021994	2.418394	not_gl.sig_2a_up	protein_co	junction adhesion molecule like
Adgrd1	0.022001	2.639262	not_gl.sig_2a_up	protein_co	adhesion G protein-coupled recep
Ccl6	0.022068	2.389772	not_gl.sig_2a_up	protein_co	chemokine (C-C motif) ligand 6
Capg	0.023433	1.58375	not_gl.sig_2a_up	protein_co	capping protein (actin filament), g
BC046401	0.023707	1.438553	not_gl.sig_2a_up	lncRNA	cDNA sequence BC046401
Med12l	0.023861	3.129666	not_gl.sig_2a_up	protein_co	mediator complex subunit 12-like
1700048O2	0.024092	2.839249	not_gl.sig_2a_up	lncRNA	RIKEN cDNA 1700048O20 gene
Heyl	0.02413	3.744214	not_gl.sig_2a_up	protein_co	hairy/enhancer-of-split related wit
Gsto1	0.024569	2.48383	not_gl.sig_2a_up	protein_co	glutathione S-transferase omega 1
Naip6	0.024721	2.563098	not_gl.sig_2a_up	protein_co	NLR family, apoptosis inhibitory pr
Qsox2	0.02554	2.074162	not_gl.sig_2a_up	protein_co	quiescin Q6 sulfhydryl oxidase 2
Exoc3l4	0.025727	2.741676	not_gl.sig_2a_up	protein_co	exocyst complex component 3-like
Bfsp2	0.025727	1.741605	not_gl.sig_2a_up	protein_co	beaded filament structural protein
AW011738	0.026057	2.304649	not_gl.sig_2a_up	lncRNA	expressed sequence AW011738
Cdh1	0.026128	2.233305	not_gl.sig_2a_up	protein_co	cadherin 1
Tbc1d4	0.026181	1.824319	not_gl.sig_2a_up	protein_co	TBC1 domain family, member 4
Nedd9	0.02636	1.262285	not_gl.sig_2a_up	protein_co	neural precursor cell expressed, de
Tpd52	0.026435	1.158482	not_gl.sig_2a_up	protein_co	tumor protein D52
Ripor2	0.026659	2.07077	not_gl.sig_2a_up	protein_co	RHO family interacting cell polariz
Diaph2	0.027144	1.780225	not_gl.sig_2a_up	protein_co	diaphanous related formin 2
Prkch	0.027227	1.265779	not_gl.sig_2a_up	protein_co	protein kinase C, eta
Podn	0.02728	2.231902	not_gl.sig_2a_up	protein_co	podocan
Gm36975	0.02728	1.640755	not_gl.sig_2a_up	lncRNA	predicted gene, 36975
Gem	0.02731	3.425129	not_gl.sig_2a_up	protein_co	GTP binding protein (gene overexp
Dipk2a	0.02731	1.237091	not_gl.sig_2a_up	protein_co	divergent protein kinase domain 2
Gbp6	0.02731	2.312466	not_gl.sig_2a_up	protein_co	guanylate binding protein 6
Pitpnc1	0.027331	1.116141	not_gl.sig_2a_up	protein_co	phosphatidylinositol transfer prote
Ube2e2	0.02737	1.951646	not_gl.sig_2a_up	protein_co	ubiquitin-conjugating enzyme E2E
Fzd7	0.02837	1.274635	not_gl.sig_2a_up	protein_co	frizzled class receptor 7
Gnb1l	0.028913	1.207739	not_gl.sig_2a_up	protein_co	guanine nucleotide binding protein
Arhgef3	0.028913	1.522168	not_gl.sig_2a_up	protein_co	Rho guanine nucleotide exchange
Dmpk	0.028913	1.611848	not_gl.sig_2a_up	protein_co	dystrophia myotonica-protein kina
Tespa1	0.028913	1.388295	not_gl.sig_2a_up	protein_co	thymocyte expressed, positive sele
Acot5	0.029152	4.250004	not_gl.sig_2a_up	protein_co	acyl-CoA thioesterase 5
Repin1	0.029152	3.59615	not_gl.sig_2a_up	protein_co	replication initiator 1
Zcchc2	0.029458	1.600695	not_gl.sig_2a_up	protein_co	zinc finger, CCHC domain containi
Prg4	0.02956	2.137367	not_gl.sig_2a_up	protein_co	proteoglycan 4 (megakaryocyte sti
Ptprv	0.02956	1.764263	not_gl.sig_2a_up	polymorph	protein tyrosine phosphatase, rece
Gm15708	0.029799	2.461845	not_gl.sig_2a_up	lncRNA	predicted gene 15708
Milr1	0.029923	1.406989	not_gl.sig_2a_up	protein_co	mast cell immunoglobulin like rece
2610307P1	0.029923	1.604443	not_gl.sig_2a_up	lncRNA	RIKEN cDNA 2610307P16 gene
Art2b	0.030212	2.716253	not_gl.sig_2a_up	protein_co	ADP-ribosyltransferase 2b
Atoh7	0.03028	3.946803	not_gl.sig_2a_up	protein_co	atonal bHLH transcription factor 7
Wdr37	0.031202	1.029089	not_gl.sig_2a_up	protein_co	WD repeat domain 37
F2rl2	0.03146	1.476161	not_gl.sig_2a_up	protein_co	coagulation factor II (thrombin) re

Wipi1	0.03146	1.722435	not_gl.sig_2a_up	protein_co	WD repeat domain, phosphoinosit
Lmcd1	0.032638	3.508116	not_gl.sig_2a_up	protein_co	LIM and cysteine-rich domains 1
Osgin1	0.032638	2.982204	not_gl.sig_2a_up	protein_co	oxidative stress induced growth in
Dapk2	0.032701	2.556533	not_gl.sig_2a_up	protein_co	death-associated protein kinase 2
Gm37881	0.033129	4.237777	not_gl.sig_2a_up	TEC	predicted gene, 37881
C130089K0	0.033129	1.759985	not_gl.sig_2a_up	TEC	RIKEN cDNA C130089K02 gene
Gimap7	0.033255	2.054708	not_gl.sig_2a_up	protein_co	GTPase, IMAP family member 7
Fam183b	0.03382	2.989636	not_gl.sig_2a_up	protein_co	family with sequence similarity 18
Card6	0.03672	1.923611	not_gl.sig_2a_up	protein_co	caspase recruitment domain famil
Gimap9	0.036863	1.106868	not_gl.sig_2a_up	protein_co	GTPase, IMAP family member 9
Gm17276	0.036932	1.364408	not_gl.sig_2a_up	lncRNA	predicted gene, 17276
Tfe3	0.036933	1.109672	not_gl.sig_2a_up	protein_co	transcription factor E3
Gm19590	0.036994	5.566779	not_gl.sig_2a_up	lncRNA	predicted gene, 19590
Gm29125	0.037444	3.964414	not_gl.sig_2a_up	lncRNA	predicted gene 29125
Ggt5	0.037604	2.245944	not_gl.sig_2a_up	protein_co	gamma-glutamyltransferase 5
Gm49274	0.037604	1.140158	not_gl.sig_2a_up	unprocesse	predicted gene, 49274
Slfn4	0.037759	3.332004	not_gl.sig_2a_up	protein_co	schlafen 4
Ryr2	0.037759	2.792207	not_gl.sig_2a_up	protein_co	ryanodine receptor 2, cardiac
Gm9973	0.037759	1.797856	not_gl.sig_2a_up	transcribec	predicted gene 9973
Itgae	0.03791	2.442889	not_gl.sig_2a_up	protein_co	integrin alpha E, epithelial-associat
Mlycd	0.038627	1.078719	not_gl.sig_2a_up	protein_co	malonyl-CoA decarboxylase
2510009E0	0.038877	1.554405	not_gl.sig_2a_up	protein_co	RIKEN cDNA 2510009E07 gene
Luzp1	0.038983	1.770216	not_gl.sig_2a_up	protein_co	leucine zipper protein 1
Gm4632	0.041543	4.340414	not_gl.sig_2a_up	lncRNA	predicted gene 4632
Spred1	0.042821	2.587798	not_gl.sig_2a_up	protein_co	sprouty protein with EVH-1 domai
Paqr5	0.043392	2.379374	not_gl.sig_2a_up	protein_co	progestin and adipoQ receptor far
Pf4	0.043796	2.001881	not_gl.sig_2a_up	protein_co	platelet factor 4
Fam43a	0.043831	2.507116	not_gl.sig_2a_up	protein_co	family with sequence similarity 43,
Rnase4	0.0468	1.89624	not_gl.sig_2a_up	protein_co	ribonuclease, RNase A family 4
Lrp12	0.047259	1.879503	not_gl.sig_2a_up	protein_co	low density lipoprotein-related pro
Sox9	0.048567	3.431755	not_gl.sig_2a_up	protein_co	SRY (sex determining region Y)-box
Zfp946	0.049761	1.391172	not_gl.sig_2a_up	protein_co	zinc finger protein 946
Hspb11	3.55E-06	-1.76566	not_gl.sig_2b_dn	protein_co	heat shock protein family B (small)
Gm46430	0.0003	-1.31709	not_gl.sig_2b_dn	lncRNA	predicted gene, 46430
Lrrc42	0.00031	-1.34921	not_gl.sig_2b_dn	protein_co	leucine rich repeat containing 42
H1f1	0.00096	-1.18657	not_gl.sig_2b_dn	protein_co	H1.1 linker histone, cluster membe
Snora30	0.003639	-1.99184	not_gl.sig_2b_dn	snoRNA	small nucleolar RNA, H/ACA box 30
H2ac22	0.003916	-1.07007	not_gl.sig_2b_dn	protein_co	H2A clustered histone 22
Rep15	0.005196	-1.44178	not_gl.sig_2b_dn	protein_co	RAB15 effector protein
Smyd1	0.010306	-3.4508	not_gl.sig_2b_dn	protein_co	SET and MYND domain containing
Mdm1	0.011832	-1.57702	not_gl.sig_2b_dn	protein_co	transformed mouse 3T3 cell doubl
Six1	0.016214	-4.88991	not_gl.sig_2b_dn	protein_co	sine oculis-related homeobox 1
Ndufb4	0.016841	-1.03862	not_gl.sig_2b_dn	protein_co	NADH:ubiquinone oxidoreductase
Syngap1	0.017457	-1.88401	not_gl.sig_2b_dn	protein_co	synaptic Ras GTPase activating pro
Ybx2	0.021277	-2.69215	not_gl.sig_2b_dn	protein_co	Y box protein 2
Vangl2	0.021277	-2.19068	not_gl.sig_2b_dn	protein_co	VANGL planar cell polarity 2
Gm4617	0.021277	-1.53474	not_gl.sig_2b_dn	processed_	predicted pseudogene 4617

Flrt1	0.021652	-2.84444	not_gl.sig_2b_dn	protein_co	fibronectin leucine rich transmeml
Gm10076	0.022955	-1.33713	not_gl.sig_2b_dn	lncRNA	predicted gene 10076
Lrrc23	0.023942	-3.58562	not_gl.sig_2b_dn	protein_co	leucine rich repeat containing 23
Chml	0.02577	-1.01715	not_gl.sig_2b_dn	protein_co	choroideremia-like
Teddm1b	0.026426	-2.24954	not_gl.sig_2b_dn	protein_co	transmembrane epididymal protei
Gm43558	0.027104	-1.26144	not_gl.sig_2b_dn	TEC	predicted gene 43558
Mns1	0.02731	-1.0718	not_gl.sig_2b_dn	protein_co	meiosis-specific nuclear structural
Sox11	0.028881	-4.13322	not_gl.sig_2b_dn	protein_co	SRY (sex determining region Y)-box
Cyb5a	0.028913	-1.21097	not_gl.sig_2b_dn	protein_co	cytochrome b5 type A (microsoma
Gm10203	0.029816	-3.7854	not_gl.sig_2b_dn	protein_co	predicted gene 10203
Gm42047	0.032188	-4.15058	not_gl.sig_2b_dn	lncRNA	predicted gene, 42047
Gm24029	0.032638	-1.84574	not_gl.sig_2b_dn	snoRNA	predicted gene, 24029
Erf	0.034576	-1.28121	not_gl.sig_2b_dn	protein_co	Ets2 repressor factor
Gm17711	0.035217	-1.20727	not_gl.sig_2b_dn	pseudogen	predicted gene, 17711
9530077C0	0.036433	-1.51529	not_gl.sig_2b_dn	protein_co	RIKEN cDNA 9530077C05 gene
Llph-ps2	0.036863	-5.02507	not_gl.sig_2b_dn	processed_	LLP homolog, pseudogene 2
Gkap1	0.037444	-1.95162	not_gl.sig_2b_dn	protein_co	G kinase anchoring protein 1
Snora75	0.037604	-1.59974	not_gl.sig_2b_dn	snoRNA	small nucleolar RNA, H/ACA box 7!
Rpusd2	0.038983	-1.02781	not_gl.sig_2b_dn	protein_co	RNA pseudouridylate synthase dor
Snora73b	0.038983	-1.44976	not_gl.sig_2b_dn	snoRNA	small nucleolar RNA, H/ACA box 7:
Scn5a	0.043363	-2.98013	not_gl.sig_2b_dn	protein_co	sodium channel, voltage-gated, ty
Gm47761	0.046098	-3.97276	not_gl.sig_2b_dn	lncRNA	predicted gene, 47761
Ubl3	0.000186	0.770984	not_gl.sig_3a_up	protein_co	ubiquitin-like 3
Srpk2	0.000568	0.896789	not_gl.sig_3a_up	protein_co	serine/arginine-rich protein specifi
Herc2	0.00152	0.555347	not_gl.sig_3a_up	protein_co	HECT and RLD domain containing I
Zswim8	0.003893	0.818852	not_gl.sig_3a_up	protein_co	zinc finger SWIM-type containing 8
Pon2	0.00444	0.927811	not_gl.sig_3a_up	protein_co	paraoxonase 2
Adcy7	0.005412	0.921714	not_gl.sig_3a_up	protein_co	adenylate cyclase 7
Mbd6	0.005467	0.827314	not_gl.sig_3a_up	protein_co	methyl-CpG binding domain prote
Pxn	0.006285	0.827085	not_gl.sig_3a_up	protein_co	paxillin
Galnt2	0.006318	0.813442	not_gl.sig_3a_up	protein_co	polypeptide N-acetylgalactosamin
Tnfaip8	0.008472	0.789443	not_gl.sig_3a_up	protein_co	tumor necrosis factor, alpha-induc
Sec16a	0.010368	0.86329	not_gl.sig_3a_up	protein_co	SEC16 homolog A, endoplasmic re
Leprot	0.010506	0.975265	not_gl.sig_3a_up	protein_co	leptin receptor overlapping transc
Rasa2	0.012756	0.880514	not_gl.sig_3a_up	protein_co	RAS p21 protein activator 2
Pgs1	0.012878	0.777851	not_gl.sig_3a_up	protein_co	phosphatidylglycerophosphate syr
Gls	0.012878	0.928438	not_gl.sig_3a_up	protein_co	glutaminase
Fbh1	0.017453	0.756135	not_gl.sig_3a_up	protein_co	F-box DNA helicase 1
Ext2	0.017925	0.738666	not_gl.sig_3a_up	protein_co	exostosin glycosyltransferase 2
Synrg	0.018178	0.578801	not_gl.sig_3a_up	protein_co	synergin, gamma
Clip1	0.018194	0.937581	not_gl.sig_3a_up	protein_co	CAP-GLY domain containing linker
Tecpr1	0.020173	0.995707	not_gl.sig_3a_up	protein_co	tectonin beta-propeller repeat cor
Ap3d1	0.02731	0.505246	not_gl.sig_3a_up	protein_co	adaptor-related protein complex 3
Mib1	0.02731	0.523955	not_gl.sig_3a_up	protein_co	mindbomb E3 ubiquitin protein lig
C1galt1c1	0.027331	0.852442	not_gl.sig_3a_up	protein_co	C1GALT1-specific chaperone 1
Etv3	0.029152	0.959875	not_gl.sig_3a_up	protein_co	ets variant 3
Vmp1	0.029439	0.933441	not_gl.sig_3a_up	protein_co	vacuole membrane protein 1

Lrp6	0.029439	0.610815	not_gl.sig_3a_up	protein_co	low density lipoprotein receptor-re
Plekhm1	0.02956	0.855152	not_gl.sig_3a_up	protein_co	pleckstrin homology domain conta
Arhgap10	0.029918	0.878731	not_gl.sig_3a_up	protein_co	Rho GTPase activating protein 10
Eml4	0.030104	0.556572	not_gl.sig_3a_up	protein_co	echinoderm microtubule associate
Pcmtd1	0.030158	0.990683	not_gl.sig_3a_up	protein_co	protein-L-isoaspartate (D-aspartat
Dedd2	0.03099	0.955227	not_gl.sig_3a_up	protein_co	death effector domain-containing
Urgcp	0.032017	0.838213	not_gl.sig_3a_up	protein_co	upregulator of cell proliferation
Gm47578	0.032951	0.992197	not_gl.sig_3a_up	lncRNA	predicted gene, 47578
Tgfbr1	0.035216	0.928758	not_gl.sig_3a_up	protein_co	transforming growth factor, beta r
Gba	0.035434	0.787366	not_gl.sig_3a_up	protein_co	glucosidase, beta, acid
Phc2	0.035434	0.579567	not_gl.sig_3a_up	protein_co	polyhomeotic 2
Crtc3	0.036011	0.719088	not_gl.sig_3a_up	protein_co	CREB regulated transcription coact
Rnf13	0.037051	0.708507	not_gl.sig_3a_up	protein_co	ring finger protein 13
Tpcn1	0.04071	0.617035	not_gl.sig_3a_up	protein_co	two pore channel 1
Evl	0.041247	0.589709	not_gl.sig_3a_up	protein_co	Ena-vasodilator stimulated phosph
Hcls1	0.041557	0.564606	not_gl.sig_3a_up	protein_co	hematopoietic cell specific Lyn sub
Npc1	0.047259	0.795876	not_gl.sig_3a_up	protein_co	NPC intracellular cholesterol trans
Mmd	0.049761	0.6566	not_gl.sig_3a_up	protein_co	monocyte to macrophage differen
Atxn7	0.049761	0.642223	not_gl.sig_3a_up	protein_co	ataxin 7
Anp32a	2.13E-07	-0.84351	not_gl.sig_3b_dn	protein_co	acidic (leucine-rich) nuclear phosp
Serf2	6.78E-06	-0.57684	not_gl.sig_3b_dn	protein_co	small EDRK-rich factor 2
H1f5	1.88E-05	-0.80926	not_gl.sig_3b_dn	protein_co	H1.5 linker histone, cluster membe
H2ac8	5.75E-05	-0.91336	not_gl.sig_3b_dn	protein_co	H2A clustered histone 8
H1f4	0.00012	-0.72015	not_gl.sig_3b_dn	protein_co	H1.4 linker histone, cluster membe
Aurka	0.00024	-0.73137	not_gl.sig_3b_dn	protein_co	aurora kinase A
Taf7	0.0003	-0.8782	not_gl.sig_3b_dn	protein_co	TATA-box binding protein associat
Dcps	0.000498	-0.65161	not_gl.sig_3b_dn	protein_co	decapping enzyme, scavenger
Bola3	0.000675	-0.63433	not_gl.sig_3b_dn	protein_co	bolA-like 3 (E. coli)
Snrpd2	0.000767	-0.64023	not_gl.sig_3b_dn	protein_co	small nuclear ribonucleoprotein D:
Erh	0.001097	-0.8069	not_gl.sig_3b_dn	protein_co	ERH mRNA splicing and mitosis fac
Tuba1c	0.00176	-0.70668	not_gl.sig_3b_dn	protein_co	tubulin, alpha 1C
Larp7	0.001867	-0.61987	not_gl.sig_3b_dn	protein_co	La ribonucleoprotein domain fami
Cks2	0.002805	-0.69674	not_gl.sig_3b_dn	protein_co	CDC28 protein kinase regulatory su
Gtf2f1	0.003084	-0.52876	not_gl.sig_3b_dn	protein_co	general transcription factor IIF, po
Wee1	0.003084	-0.74298	not_gl.sig_3b_dn	protein_co	WEE 1 homolog 1 (S. pombe)
Sarnp	0.003422	-0.60323	not_gl.sig_3b_dn	protein_co	SAP domain containing ribonucleo
Snrpd1	0.003524	-0.68066	not_gl.sig_3b_dn	protein_co	small nuclear ribonucleoprotein D:
Supt4a	0.00356	-0.68084	not_gl.sig_3b_dn	protein_co	SPT4A, DSIF elongation factor subu
Lsm5	0.003916	-0.59142	not_gl.sig_3b_dn	protein_co	LSM5 homolog, U6 small nuclear R
Fh1	0.004358	-0.83205	not_gl.sig_3b_dn	protein_co	fumarate hydratase 1
Bub1b	0.004358	-0.63005	not_gl.sig_3b_dn	protein_co	BUB1B, mitotic checkpoint serine/
Atp5j	0.004881	-0.59178	not_gl.sig_3b_dn	protein_co	ATP synthase, H+ transporting, mit
Cnot2	0.004899	-0.74535	not_gl.sig_3b_dn	protein_co	CCR4-NOT transcription complex, s
Lsm12	0.005467	-0.84409	not_gl.sig_3b_dn	protein_co	LSM12 homolog
Lcmt1	0.006285	-0.63841	not_gl.sig_3b_dn	protein_co	leucine carboxyl methyltransferase
Ncl	0.006497	-0.60922	not_gl.sig_3b_dn	protein_co	nucleolin
Gm17134	0.007016	-0.5854	not_gl.sig_3b_dn	lncRNA	predicted gene 17134

Sdhb	0.007604	-0.59768	not_gl.sig_3b_dn	protein_co	succinate dehydrogenase complex
Leo1	0.009834	-0.8477	not_gl.sig_3b_dn	protein_co	Leo1, Paf1/RNA polymerase II com
Ino80b	0.010506	-0.90948	not_gl.sig_3b_dn	protein_co	INO80 complex subunit B
Pfdn4	0.010674	-0.63746	not_gl.sig_3b_dn	protein_co	prefoldin 4
Lmnb1	0.011055	-0.72294	not_gl.sig_3b_dn	protein_co	lamin B1
Snrpd3	0.011154	-0.60622	not_gl.sig_3b_dn	protein_co	small nuclear ribonucleoprotein D
Pfdn6	0.011622	-0.65718	not_gl.sig_3b_dn	protein_co	prefoldin subunit 6
Rpl36-ps12	0.014685	-0.62226	not_gl.sig_3b_dn	processed_	ribosomal protein L36, pseudogen
Lsm7	0.01494	-0.58589	not_gl.sig_3b_dn	protein_co	LSM7 homolog, U6 small nuclear F
Hmmr	0.015888	-0.68036	not_gl.sig_3b_dn	protein_co	hyaluronan mediated motility rece
Selenoi	0.016208	-0.94225	not_gl.sig_3b_dn	protein_co	selenoprotein I
Zw10	0.016228	-0.73663	not_gl.sig_3b_dn	protein_co	zw10 kinetochore protein
Pfdn2	0.016307	-0.55837	not_gl.sig_3b_dn	protein_co	prefoldin 2
Strbp	0.016307	-0.88378	not_gl.sig_3b_dn	protein_co	spermatid perinuclear RNA binding
Mrps22	0.017499	-0.63346	not_gl.sig_3b_dn	protein_co	mitochondrial ribosomal protein S
Rraga	0.018019	-0.60254	not_gl.sig_3b_dn	protein_co	Ras-related GTP binding A
Gm13577	0.018019	-0.65868	not_gl.sig_3b_dn	processed_	predicted gene 13577
Kif4	0.018108	-0.66161	not_gl.sig_3b_dn	protein_co	kinesin family member 4
Dpy30	0.018153	-0.67684	not_gl.sig_3b_dn	protein_co	dpy-30, histone methyltransferase
Mrpl13	0.018178	-0.74237	not_gl.sig_3b_dn	protein_co	mitochondrial ribosomal protein L
Khsrp	0.018474	-0.54982	not_gl.sig_3b_dn	protein_co	KH-type splicing regulatory proteir
Pgls	0.018474	-0.66481	not_gl.sig_3b_dn	protein_co	6-phosphogluconolactonase
Clic1	0.018866	-0.58954	not_gl.sig_3b_dn	protein_co	chloride intracellular channel 1
Hmgb2	0.020173	-0.61144	not_gl.sig_3b_dn	protein_co	high mobility group box 2
Atl2	0.020911	-0.7988	not_gl.sig_3b_dn	protein_co	atlastin GTPase 2
Xrcc1	0.021134	-0.66386	not_gl.sig_3b_dn	protein_co	X-ray repair complementing defec
Fuz	0.022384	-0.87856	not_gl.sig_3b_dn	protein_co	fuzzy planar cell polarity protein
Aarsd1	0.022955	-0.71087	not_gl.sig_3b_dn	protein_co	alanyl-tRNA synthetase domain co
Gmnn	0.026353	-0.61075	not_gl.sig_3b_dn	protein_co	geminin
Stip1	0.02728	-0.65671	not_gl.sig_3b_dn	protein_co	stress-induced phosphoprotein 1
Prpf19	0.02731	-0.68689	not_gl.sig_3b_dn	protein_co	pre-mRNA processing factor 19
Atp5g2	0.02731	-0.72798	not_gl.sig_3b_dn	protein_co	ATP synthase, H+ transporting, mi
Rpf2	0.028913	-0.62533	not_gl.sig_3b_dn	protein_co	ribosome production factor 2 hom
Polr2j	0.029152	-0.51531	not_gl.sig_3b_dn	protein_co	polymerase (RNA) II (DNA directec
Lbr	0.029458	-0.50577	not_gl.sig_3b_dn	protein_co	lamin B receptor
Smarcd2	0.029458	-0.51103	not_gl.sig_3b_dn	protein_co	SWI/SNF related, matrix associate
Ppih	0.030053	-0.5776	not_gl.sig_3b_dn	protein_co	peptidyl prolyl isomerase H
Nusap1	0.030113	-0.61637	not_gl.sig_3b_dn	protein_co	nucleolar and spindle associated p
Senp3	0.030158	-0.5741	not_gl.sig_3b_dn	protein_co	SUMO/sentrin specific peptidase 3
4930453N2	0.030748	-0.77165	not_gl.sig_3b_dn	protein_co	RIKEN cDNA 4930453N24 gene
Shcbp1	0.03189	-0.62463	not_gl.sig_3b_dn	protein_co	Shc SH2-domain binding protein 1
Rpa2	0.032017	-0.60992	not_gl.sig_3b_dn	protein_co	replication protein A2
Eif3j2	0.032638	-0.76509	not_gl.sig_3b_dn	protein_co	eukaryotic translation initiation fa
Rpl36al	0.032638	-0.52405	not_gl.sig_3b_dn	protein_co	ribosomal protein L36A-like
Nsmce2	0.032951	-0.69493	not_gl.sig_3b_dn	protein_co	NSE2/MMS21 homolog, SMC5-SM
Pola1	0.033087	-0.54289	not_gl.sig_3b_dn	protein_co	polymerase (DNA directed), alpha
Snora81	0.033255	-0.99557	not_gl.sig_3b_dn	snoRNA	small nucleolar RNA, H/ACA box 8

Cdc20	0.033725	-0.53542	not_gl.sig_3b_dn	protein_co	cell division cycle 20
Ndufa4	0.03382	-0.5334	not_gl.sig_3b_dn	protein_co	Ndufa4, mitochondrial complex as
Lsm4	0.034687	-0.59575	not_gl.sig_3b_dn	protein_co	LSM4 homolog, U6 small nuclear F
Rpa3	0.037604	-0.67344	not_gl.sig_3b_dn	protein_co	replication protein A3
Ndufa5	0.03791	-0.66054	not_gl.sig_3b_dn	protein_co	NADH:ubiquinone oxidoreductase
Aplf	0.038087	-0.88683	not_gl.sig_3b_dn	protein_co	aprataxin and PNKP like factor
Nudc	0.038627	-0.58917	not_gl.sig_3b_dn	protein_co	nudC nuclear distribution protein
Zfp955a	0.03898	-0.80797	not_gl.sig_3b_dn	protein_co	zinc finger protein 955A
Bhlhb9	0.038983	-0.70408	not_gl.sig_3b_dn	protein_co	basic helix-loop-helix domain cont
Npm3	0.040333	-0.54347	not_gl.sig_3b_dn	protein_co	nucleoplasmin 3
Mrpl50	0.040534	-0.66804	not_gl.sig_3b_dn	protein_co	mitochondrial ribosomal protein L
Snord89	0.042127	-0.84615	not_gl.sig_3b_dn	snoRNA	small nucleolar RNA, C/D box 89
Pdik1l	0.043715	-0.50747	not_gl.sig_3b_dn	protein_co	PDLIM1 interacting kinase 1 like
Kif20b	0.043831	-0.57868	not_gl.sig_3b_dn	protein_co	kinesin family member 20B
Mtx1	0.04653	-0.52418	not_gl.sig_3b_dn	protein_co	metaxin 1
Mfap5	0.050015	1.814967	not_gl.sig_4a_up	protein_co	microfibrillar associated protein 5
Ccdc125	0.05018	1.097896	not_gl.sig_4a_up	protein_co	coiled-coil domain containing 125
Phf11d	0.050342	1.269648	not_gl.sig_4a_up	protein_co	PHD finger protein 11D
C030005K0	0.050342	1.404374	not_gl.sig_4a_up	lncRNA	RIKEN cDNA C030005K06 gene
Nav1	0.050761	1.278867	not_gl.sig_4a_up	protein_co	neuron navigator 1
Tspan13	0.051563	1.686463	not_gl.sig_4a_up	protein_co	tetraspanin 13
Tmcc1	0.051706	0.760109	not_gl.sig_4a_up	protein_co	transmembrane and coiled coil do
A430027C0	0.051768	0.892041	not_gl.sig_4a_up	TEC	RIKEN cDNA A430027C01 gene
Pmepa1	0.051921	1.080646	not_gl.sig_4a_up	protein_co	prostate transmembrane protein,
5830418P1	0.052429	1.651194	not_gl.sig_4a_up	lncRNA	RIKEN cDNA 5830418P13 gene
Tpst2	0.052568	0.785649	not_gl.sig_4a_up	protein_co	protein-tyrosine sulfotransferase 2
Rbm47	0.052568	1.679523	not_gl.sig_4a_up	protein_co	RNA binding motif protein 47
Gm44189	0.052568	1.023777	not_gl.sig_4a_up	TEC	predicted gene, 44189
Kremen1	0.052663	0.872796	not_gl.sig_4a_up	protein_co	kringle containing transmembrane
Rdh10	0.052967	2.107821	not_gl.sig_4a_up	protein_co	retinol dehydrogenase 10 (all-tran
Rnf150	0.052967	1.622904	not_gl.sig_4a_up	protein_co	ring finger protein 150
Sertad4	0.05361	2.732857	not_gl.sig_4a_up	protein_co	SERTA domain containing 4
Rap1gds1	0.05361	0.708631	not_gl.sig_4a_up	protein_co	RAP1, GTP-GDP dissociation stimu
Sema7a	0.05361	1.863269	not_gl.sig_4a_up	protein_co	sema domain, immunoglobulin do
Gbf1	0.053685	1.181495	not_gl.sig_4a_up	protein_co	golgi-specific brefeldin A-resistanc
Phlpp1	0.053685	1.869279	not_gl.sig_4a_up	protein_co	PH domain and leucine rich repeat
Usp12	0.053925	0.553428	not_gl.sig_4a_up	protein_co	ubiquitin specific peptidase 12
Firre	0.053925	1.586599	not_gl.sig_4a_up	lncRNA	functional intergenic repeating RN
Mafb	0.053963	1.080167	not_gl.sig_4a_up	protein_co	v-maf musculoaponeurotic fibrosa
Alox5ap	0.055133	1.956783	not_gl.sig_4a_up	protein_co	arachidonate 5-lipoxygenase activa
Ctla2b	0.055251	1.920538	not_gl.sig_4a_up	protein_co	cytotoxic T lymphocyte-associated
St3gal1	0.056603	1.299241	not_gl.sig_4a_up	protein_co	ST3 beta-galactoside alpha-2,3-sia
Bag3	0.056603	1.408357	not_gl.sig_4a_up	protein_co	BCL2-associated athanogene 3
Inafm1	0.05677	0.931591	not_gl.sig_4a_up	protein_co	InaF motif containing 1
Zfp993	0.057424	1.358543	not_gl.sig_4a_up	protein_co	zinc finger protein 993
Zfp641	0.058204	5.377718	not_gl.sig_4a_up	protein_co	zinc finger protein 641
Etv6	0.058204	1.028013	not_gl.sig_4a_up	protein_co	ets variant 6

Tcf12	0.058204	0.580013	not_gl.sig_4a_up	protein_co	transcription factor 12
Elmod2	0.058204	1.210661	not_gl.sig_4a_up	protein_co	ELMO/CED-12 domain containing
Ncald	0.058375	1.966653	not_gl.sig_4a_up	protein_co	neurocalcin delta
Dtx1	0.058629	3.431405	not_gl.sig_4a_up	protein_co	deltex 1, E3 ubiquitin ligase
Fam222b	0.058629	0.877737	not_gl.sig_4a_up	protein_co	family with sequence similarity 22
Cdk5rap2	0.058629	1.15855	not_gl.sig_4a_up	protein_co	CDK5 regulatory subunit associate
Gm42900	0.058629	0.908544	not_gl.sig_4a_up	TEC	predicted gene 42900
Twsg1	0.059679	0.762788	not_gl.sig_4a_up	protein_co	twisted gastrulation BMP signaling
Pag1	0.059679	1.377808	not_gl.sig_4a_up	protein_co	phosphoprotein associated with gl
Vasn	0.059819	1.198344	not_gl.sig_4a_up	protein_co	vasorin
Pakap	0.059879	1.399893	not_gl.sig_4a_up	protein_co	paralemmin A kinase anchor prote
Sbno2	0.059931	0.560879	not_gl.sig_4a_up	protein_co	strawberry notch 2
Parp8	0.06044	1.705032	not_gl.sig_4a_up	protein_co	poly (ADP-ribose) polymerase fam
Rsu1	0.06044	1.040835	not_gl.sig_4a_up	protein_co	Ras suppressor protein 1
Lix1l	0.060678	1.084013	not_gl.sig_4a_up	protein_co	Lix1-like
Gimap5	0.060888	1.585554	not_gl.sig_4a_up	protein_co	GTPase, IMAP family member 5
Rflnb	0.061229	1.633158	not_gl.sig_4a_up	protein_co	refilin B
Pde10a	0.061229	2.676701	not_gl.sig_4a_up	protein_co	phosphodiesterase 10A
Lpp	0.061229	1.22226	not_gl.sig_4a_up	protein_co	LIM domain containing preferred t
Nlrc3	0.061229	0.750241	not_gl.sig_4a_up	protein_co	NLR family, CARD domain containi
S100a4	0.061585	1.678759	not_gl.sig_4a_up	protein_co	S100 calcium binding protein A4
Tbc1d22b	0.062435	0.813059	not_gl.sig_4a_up	protein_co	TBC1 domain family, member 22B
Cd248	0.062435	1.961327	not_gl.sig_4a_up	protein_co	CD248 antigen, endosialin
Nkd1	0.06247	2.948829	not_gl.sig_4a_up	protein_co	naked cuticle 1
Cuedc1	0.062572	1.097938	not_gl.sig_4a_up	protein_co	CUE domain containing 1
Gm12216	0.062572	1.806731	not_gl.sig_4a_up	protein_co	predicted gene 12216
Galnt2l	0.062572	0.718907	not_gl.sig_4a_up	protein_co	polypeptide N-acetylgalactosamin
AB124611	0.063697	0.970502	not_gl.sig_4a_up	protein_co	cDNA sequence AB124611
Dusp10	0.063889	1.830432	not_gl.sig_4a_up	protein_co	dual specificity phosphatase 10
Apbb2	0.064117	1.850987	not_gl.sig_4a_up	protein_co	amyloid beta (A4) precursor prote
Gimap1	0.064313	1.914841	not_gl.sig_4a_up	protein_co	GTPase, IMAP family member 1
Usp20	0.064415	1.038726	not_gl.sig_4a_up	protein_co	ubiquitin specific peptidase 20
Gnao1	0.064415	3.48105	not_gl.sig_4a_up	protein_co	guanine nucleotide binding protein
Ngfr	0.064452	3.820187	not_gl.sig_4a_up	protein_co	nerve growth factor receptor (TNF
Itm2c	0.064524	0.801601	not_gl.sig_4a_up	protein_co	integral membrane protein 2C
Mrc1	0.064524	1.687561	not_gl.sig_4a_up	protein_co	mannose receptor, C type 1
Dock10	0.064524	1.3711	not_gl.sig_4a_up	protein_co	dedicator of cytokinesis 10
Gda	0.064524	1.715975	not_gl.sig_4a_up	protein_co	guanine deaminase
Akr1c13	0.06453	2.65736	not_gl.sig_4a_up	protein_co	aldo-keto reductase family 1, mem
Clcn5	0.064615	0.820728	not_gl.sig_4a_up	protein_co	chloride channel, voltage-sensitive
Ackr1	0.064615	2.038393	not_gl.sig_4a_up	protein_co	atypical chemokine receptor 1 (Du
Cmtm3	0.064762	1.018104	not_gl.sig_4a_up	protein_co	CKLF-like MARVEL transmembrane
Myl4	0.064762	2.552823	not_gl.sig_4a_up	protein_co	myosin, light polypeptide 4
Oasl2	0.065125	1.74209	not_gl.sig_4a_up	protein_co	2'-5' oligoadenylate synthetase-lik
Epas1	0.065267	1.276073	not_gl.sig_4a_up	protein_co	endothelial PAS domain protein 1
Dennd1a	0.065267	0.587439	not_gl.sig_4a_up	protein_co	DENN/MADD domain containing 1
5330426L2	0.065267	3.090006	not_gl.sig_4a_up	lncRNA	RIKEN cDNA 5330426L24 gene

Sgk1	0.06558	1.33834	not_gl.sig_4a_up	protein_co	serum/glucocorticoid regulated ki
Mta1	0.06558	0.539585	not_gl.sig_4a_up	protein_co	metastasis associated 1
Khdc1a	0.06558	6.128341	not_gl.sig_4a_up	protein_co	KH domain containing 1A
Agfg1	0.06579	2.039232	not_gl.sig_4a_up	protein_co	ArfGAP with FG repeats 1
Septin9	0.065847	0.606474	not_gl.sig_4a_up	protein_co	septin 9
Lhx2	0.066306	3.82251	not_gl.sig_4a_up	protein_co	LIM homeobox protein 2
Ccr1	0.066464	2.723696	not_gl.sig_4a_up	protein_co	chemokine (C-C motif) receptor 1
Zbtb10	0.067055	3.923641	not_gl.sig_4a_up	protein_co	zinc finger and BTB domain contai
Gm13568	0.067055	2.607955	not_gl.sig_4a_up	lncRNA	predicted gene 13568
Stard8	0.067209	1.806761	not_gl.sig_4a_up	protein_co	START domain containing 8
Rhoj	0.067408	1.445056	not_gl.sig_4a_up	protein_co	ras homolog family member J
Gm44739	0.067593	3.188801	not_gl.sig_4a_up	lncRNA	predicted gene 44739
Snrk	0.068287	1.133212	not_gl.sig_4a_up	protein_co	SNF related kinase
Ptprk	0.068327	1.679658	not_gl.sig_4a_up	protein_co	protein tyrosine phosphatase, rece
Ap2a1	0.068567	0.722675	not_gl.sig_4a_up	protein_co	adaptor-related protein complex 2
Ly6c1	0.068567	2.131824	not_gl.sig_4a_up	protein_co	lymphocyte antigen 6 complex, loc
Gbp8	0.068579	2.033172	not_gl.sig_4a_up	protein_co	guanylate-binding protein 8
Fhl1	0.069158	1.96169	not_gl.sig_4a_up	protein_co	four and a half LIM domains 1
Cpd	0.069659	1.692748	not_gl.sig_4a_up	protein_co	carboxypeptidase D
Pdlim7	0.069904	1.1378	not_gl.sig_4a_up	protein_co	PDZ and LIM domain 7
Gm15655	0.070093	1.911948	not_gl.sig_4a_up	lncRNA	predicted gene 15655
Jag1	0.070192	2.038113	not_gl.sig_4a_up	protein_co	jagged 1
Gtpbp2	0.070862	0.722465	not_gl.sig_4a_up	protein_co	GTP binding protein 2
Irgm1	0.071628	1.060381	not_gl.sig_4a_up	protein_co	immunity-related GTPase family M
Tor3a	0.071911	1.007444	not_gl.sig_4a_up	protein_co	torsin family 3, member A
Unc119	0.073683	1.194527	not_gl.sig_4a_up	protein_co	unc-119 lipid binding chaperone
Fam163b	0.073683	5.170869	not_gl.sig_4a_up	protein_co	family with sequence similarity 16
Aldh1b1	0.073683	3.23377	not_gl.sig_4a_up	protein_co	aldehyde dehydrogenase 1 family,
Slc45a4	0.073683	0.776947	not_gl.sig_4a_up	protein_co	solute carrier family 45, member 4
Mirt1	0.073683	1.820106	not_gl.sig_4a_up	lncRNA	myocardial infarction associated tr
Plekhg3	0.073725	1.893726	not_gl.sig_4a_up	protein_co	pleckstrin homology domain conta
Nfia	0.073932	0.904386	not_gl.sig_4a_up	protein_co	nuclear factor I/A
Brdt	0.074007	1.89964	not_gl.sig_4a_up	protein_co	bromodomain, testis-specific
Agpat3	0.074413	0.613208	not_gl.sig_4a_up	protein_co	1-acylglycerol-3-phosphate O-acyl
Aebp1	0.074569	1.664913	not_gl.sig_4a_up	protein_co	AE binding protein 1
Bin1	0.074577	1.513839	not_gl.sig_4a_up	protein_co	bridging integrator 1
Asph	0.074577	1.890084	not_gl.sig_4a_up	protein_co	aspartate-beta-hydroxylase
Rab8b	0.074684	0.781733	not_gl.sig_4a_up	protein_co	RAB8B, member RAS oncogene fa
Niban2	0.074703	1.248459	not_gl.sig_4a_up	protein_co	niban apoptosis regulator 2
Ttc7	0.074703	0.610789	not_gl.sig_4a_up	protein_co	tetratricopeptide repeat domain 7
Gpx3	0.075172	1.728334	not_gl.sig_4a_up	protein_co	glutathione peroxidase 3
Dtx3l	0.075172	0.878583	not_gl.sig_4a_up	protein_co	deltex 3-like, E3 ubiquitin ligase
Gm38198	0.075172	1.487879	not_gl.sig_4a_up	TEC	predicted gene, 38198
Pdk4	0.07721	1.738323	not_gl.sig_4a_up	protein_co	pyruvate dehydrogenase kinase, is
Emp1	0.078137	1.232455	not_gl.sig_4a_up	protein_co	epithelial membrane protein 1
Bzw2	0.078447	0.752642	not_gl.sig_4a_up	protein_co	basic leucine zipper and W2 doma
Pcnx	0.078447	1.023418	not_gl.sig_4a_up	protein_co	pecanex homolog

Plod2	0.078447	2.801995	not_gl.sig_4a_up	protein_co	procollagen lysine, 2-oxoglutarate
Wfs1	0.078447	1.419108	not_gl.sig_4a_up	protein_co	wolframin ER transmembrane glyc
Gm48082	0.078447	1.094254	not_gl.sig_4a_up	processed_	predicted gene, 48082
Kcnj8	0.07858	1.980413	not_gl.sig_4a_up	protein_co	potassium inwardly-rectifying chai
Snx13	0.07871	0.844027	not_gl.sig_4a_up	protein_co	sorting nexin 13
Map3k5	0.080198	1.740448	not_gl.sig_4a_up	protein_co	mitogen-activated protein kinase k
Castor1	0.0802	2.158283	not_gl.sig_4a_up	protein_co	cytosolic arginine sensor for mTOF
Mettl6	0.0802	0.823465	not_gl.sig_4a_up	protein_co	methyltransferase like 6
Angpt1	0.0802	2.432594	not_gl.sig_4a_up	protein_co	angiopoietin 1
Ablim1	0.080384	0.672533	not_gl.sig_4a_up	protein_co	actin-binding LIM protein 1
Sestd1	0.081678	2.029475	not_gl.sig_4a_up	protein_co	SEC14 and spectrin domains 1
Stard3nl	0.081692	1.130982	not_gl.sig_4a_up	protein_co	STARD3 N-terminal like
Nucb1	0.081692	0.71235	not_gl.sig_4a_up	protein_co	nucleobindin 1
Spef2	0.081692	2.976838	not_gl.sig_4a_up	protein_co	sperm flagellar 2
Gm43336	0.081692	0.81303	not_gl.sig_4a_up	TEC	predicted gene 43336
Col16a1	0.08216	1.993511	not_gl.sig_4a_up	protein_co	collagen, type XVI, alpha 1
Ptprf	0.082664	1.012765	not_gl.sig_4a_up	protein_co	protein tyrosine phosphatase, rece
Gimap4	0.082664	1.976558	not_gl.sig_4a_up	protein_co	GTPase, IMAP family member 4
Kif13a	0.082875	1.771469	not_gl.sig_4a_up	protein_co	kinesin family member 13A
Saa3	0.082902	2.11516	not_gl.sig_4a_up	protein_co	serum amyloid A 3
Cmas	0.083255	0.653828	not_gl.sig_4a_up	protein_co	cytidine monophospho-N-acetylne
Zfp362	0.083383	0.782967	not_gl.sig_4a_up	protein_co	zinc finger protein 362
Tenm1	0.083737	2.050201	not_gl.sig_4a_up	protein_co	teneurin transmembrane protein 1
Mafg	0.083737	0.636778	not_gl.sig_4a_up	protein_co	v-maf musculoaponeurotic fibrosa
Tmem86a	0.083863	1.171686	not_gl.sig_4a_up	protein_co	transmembrane protein 86A
Plk2	0.084054	2.215857	not_gl.sig_4a_up	protein_co	polo like kinase 2
Gmds	0.084637	0.772246	not_gl.sig_4a_up	protein_co	GDP-mannose 4, 6-dehydratase
Plekha7	0.084637	1.798811	not_gl.sig_4a_up	protein_co	pleckstrin homology domain conta
Rhoq	0.08484	0.72644	not_gl.sig_4a_up	protein_co	ras homolog family member Q
Gm37124	0.084937	1.166942	not_gl.sig_4a_up	TEC	predicted gene, 37124
Crip2	0.085209	0.894503	not_gl.sig_4a_up	protein_co	cysteine rich protein 2
Anapc11	0.085961	0.568558	not_gl.sig_4a_up	protein_co	anaphase promoting complex subu
Abhd4	0.08613	1.235311	not_gl.sig_4a_up	protein_co	abhydrolase domain containing 4
Olfr1487	0.08613	2.341325	not_gl.sig_4a_up	protein_co	olfactory receptor 1487
Hspb1	0.086596	1.421388	not_gl.sig_4a_up	protein_co	heat shock protein 1
F830016B0	0.086743	0.995565	not_gl.sig_4a_up	protein_co	RIKEN cDNA F830016B08 gene
Gm47015	0.086743	1.488302	not_gl.sig_4a_up	lncRNA	predicted gene, 47015
Clec4a2	0.087186	1.463073	not_gl.sig_4a_up	protein_co	C-type lectin domain family 4, mer
Cebpb	0.087186	0.970953	not_gl.sig_4a_up	protein_co	CCAAT/enhancer binding protein (
Galnt10	0.087728	1.423487	not_gl.sig_4a_up	protein_co	polypeptide N-acetylgalactosamin
Gm47248	0.088025	1.566629	not_gl.sig_4a_up	lncRNA	predicted gene, 47248
Fads3	0.088504	2.316423	not_gl.sig_4a_up	protein_co	fatty acid desaturase 3
Tmem119	0.088504	1.916009	not_gl.sig_4a_up	protein_co	transmembrane protein 119
Herpud2	0.088507	0.563282	not_gl.sig_4a_up	protein_co	HERPUD family member 2
Cdc42ep4	0.088549	1.550252	not_gl.sig_4a_up	protein_co	CDC42 effector protein (Rho GTPa
Elfn2	0.089167	2.845516	not_gl.sig_4a_up	protein_co	leucine rich repeat and fibronectin
Osbpl1a	0.089206	1.792041	not_gl.sig_4a_up	protein_co	oxysterol binding protein-like 1A

Insig2	0.089243	0.78069	not_gl.sig_4a_up	protein_co	insulin induced gene 2
Acy3	0.089882	3.375838	not_gl.sig_4a_up	protein_co	aspartoacylase (aminoacylase) 3
Man2b2	0.089961	0.719337	not_gl.sig_4a_up	protein_co	mannosidase 2, alpha B2
Wdr33	0.090235	0.589458	not_gl.sig_4a_up	protein_co	WD repeat domain 33
Bivm	0.090235	1.468578	not_gl.sig_4a_up	protein_co	basic, immunoglobulin-like variabl
Tmem121	0.090235	2.857142	not_gl.sig_4a_up	protein_co	transmembrane protein 121
Trim30d	0.091193	1.671404	not_gl.sig_4a_up	protein_co	tripartite motif-containing 30D
Neb	0.091205	1.703441	not_gl.sig_4a_up	protein_co	nebulin
Mknk1	0.091205	0.559374	not_gl.sig_4a_up	protein_co	MAP kinase-interacting serine/thr
Cnga3	0.091248	1.135993	not_gl.sig_4a_up	protein_co	cyclic nucleotide gated channel alp
Tk2	0.091248	0.686939	not_gl.sig_4a_up	protein_co	thymidine kinase 2, mitochondrial
Phldb1	0.091248	1.952971	not_gl.sig_4a_up	protein_co	pleckstrin homology like domain, f
Gm15856	0.091248	1.481223	not_gl.sig_4a_up	lncRNA	predicted gene 15856
Gm43238	0.091248	0.878064	not_gl.sig_4a_up	TEC	predicted gene 43238
Gm44060	0.091847	1.94015	not_gl.sig_4a_up	processed_	predicted gene, 44060
Tep1	0.092055	0.803324	not_gl.sig_4a_up	protein_co	telomerase associated protein 1
Thnsl2	0.092059	1.233417	not_gl.sig_4a_up	protein_co	threonine synthase-like 2 (bacteria
Nsmf	0.092498	1.377717	not_gl.sig_4a_up	protein_co	NMDA receptor synaptonuclear si
Mgp	0.092556	1.263127	not_gl.sig_4a_up	protein_co	matrix Gla protein
Dennd2d	0.093032	1.89804	not_gl.sig_4a_up	protein_co	DENN/MADD domain containing 2
Ndst1	0.09316	0.732048	not_gl.sig_4a_up	protein_co	N-deacetylase/N-sulfotransferase
Zcchc24	0.093895	1.116898	not_gl.sig_4a_up	protein_co	zinc finger, CCHC domain containi
Fbln1	0.094938	0.830922	not_gl.sig_4a_up	protein_co	fibulin 1
Tmem241	0.094938	0.947683	not_gl.sig_4a_up	protein_co	transmembrane protein 241
Med7	0.095189	0.7797	not_gl.sig_4a_up	protein_co	mediator complex subunit 7
Smpd3	0.095189	1.420515	not_gl.sig_4a_up	protein_co	sphingomyelin phosphodiesterase
Gm39312	0.096067	0.868413	not_gl.sig_4a_up	lncRNA	predicted gene, 39312
Pla2g7	0.096244	1.517311	not_gl.sig_4a_up	protein_co	phospholipase A2, group VII (plate
Col5a2	0.097364	1.683298	not_gl.sig_4a_up	protein_co	collagen, type V, alpha 2
Ctla4	0.097417	3.160538	not_gl.sig_4a_up	protein_co	cytotoxic T-lymphocyte-associatec
Dst	0.097838	1.320559	not_gl.sig_4a_up	protein_co	dystonin
Tgfbi	0.098176	1.634298	not_gl.sig_4a_up	protein_co	transforming growth factor, beta i
Cemip2	0.098331	1.237814	not_gl.sig_4a_up	protein_co	cell migration inducing hyaluronid
Fstl1	0.098494	1.291661	not_gl.sig_4a_up	protein_co	follistatin-like 1
Ptcra	0.098494	1.911314	not_gl.sig_4a_up	protein_co	pre T cell antigen receptor alpha
Myo7a	0.098683	1.499339	not_gl.sig_4a_up	protein_co	myosin VIIA
Wwp1	0.098683	0.665312	not_gl.sig_4a_up	protein_co	WW domain containing E3 ubiquit
Pid1	0.098683	1.524355	not_gl.sig_4a_up	protein_co	phosphotyrosine interaction doma
Vopp1	0.099591	1.158702	not_gl.sig_4a_up	protein_co	vesicular, overexpressed in cancer
Cebpa	0.09987	1.38284	not_gl.sig_4a_up	protein_co	CCAAT/enhancer binding protein (
Mcm7	0.050899	-0.57598	not_gl.sig_4b_dn	protein_co	minichromosome maintenance co
Mrps36	0.050899	-0.63765	not_gl.sig_4b_dn	protein_co	mitochondrial ribosomal protein S
Mr1	0.05098	-1.3614	not_gl.sig_4b_dn	protein_co	major histocompatibility complex,
Gm38312	0.05108	-1.31788	not_gl.sig_4b_dn	TEC	predicted gene, 38312
Trub1	0.05143	-0.67311	not_gl.sig_4b_dn	protein_co	TruB pseudouridine (psi) synthase
Snrpg	0.05143	-0.55708	not_gl.sig_4b_dn	protein_co	small nuclear ribonucleoprotein p
Ssx2ip	0.051768	-1.36298	not_gl.sig_4b_dn	protein_co	synovial sarcoma, X 2 interacting p

Pi4kb	0.051768	-0.6973	not_gl.sig_4b_dn	protein_co	phosphatidylinositol 4-kinase beta
Cip2a	0.051857	-0.54325	not_gl.sig_4b_dn	protein_co	cell proliferation regulating inhibit
Dap3	0.052093	-0.54185	not_gl.sig_4b_dn	protein_co	death associated protein 3
Pin4	0.052378	-0.66946	not_gl.sig_4b_dn	protein_co	protein (peptidyl-prolyl cis/trans is
Tuba1b	0.052568	-0.64126	not_gl.sig_4b_dn	protein_co	tubulin, alpha 1B
Fn3krp	0.052568	-0.94846	not_gl.sig_4b_dn	protein_co	fructosamine 3 kinase related prot
Ckap2l	0.052568	-0.59947	not_gl.sig_4b_dn	protein_co	cytoskeleton associated protein 2-
Rbm38	0.052592	-0.79001	not_gl.sig_4b_dn	protein_co	RNA binding motif protein 38
Rpf1	0.052592	-0.5484	not_gl.sig_4b_dn	protein_co	ribosome production factor 1 hom
Gm43055	0.052967	-0.7071	not_gl.sig_4b_dn	lncRNA	predicted gene 43055
1810013D1	0.053835	-0.76555	not_gl.sig_4b_dn	lncRNA	RIKEN cDNA 1810013D15 gene
Exosc8	0.053963	-0.58303	not_gl.sig_4b_dn	protein_co	exosome component 8
Rcbtb2	0.054386	-1.10549	not_gl.sig_4b_dn	protein_co	regulator of chromosome condens
Nop10	0.056979	-0.64422	not_gl.sig_4b_dn	protein_co	NOP10 ribonucleoprotein
Snhg9	0.057318	-0.69112	not_gl.sig_4b_dn	lncRNA	small nucleolar RNA host gene 9
Palb2	0.057395	-0.58565	not_gl.sig_4b_dn	protein_co	partner and localizer of BRCA2
Hmga1	0.058204	-0.61541	not_gl.sig_4b_dn	protein_co	high mobility group AT-hook 1
5830417I1	0.058629	-1.21471	not_gl.sig_4b_dn	unprocesse	RIKEN cDNA 5830417I10 gene
Fignl1	0.059679	-0.61986	not_gl.sig_4b_dn	protein_co	fidgetin-like 1
Gpr162	0.06044	-2.06494	not_gl.sig_4b_dn	protein_co	G protein-coupled receptor 162
Mmgt1	0.06044	-0.66062	not_gl.sig_4b_dn	protein_co	membrane magnesium transporte
Gm21596	0.06044	-0.61913	not_gl.sig_4b_dn	processed_	predicted gene, 21596
Pvt1	0.06044	-1.97296	not_gl.sig_4b_dn	lncRNA	Pvt1 oncogene
Nr1d1	0.060614	-1.94489	not_gl.sig_4b_dn	protein_co	nuclear receptor subfamily 1, grou
Ssbp4	0.061229	-0.53444	not_gl.sig_4b_dn	protein_co	single stranded DNA binding prote
H2ac24	0.062147	-0.5104	not_gl.sig_4b_dn	protein_co	H2A clustered histone 24
Sh3gl3	0.062572	-2.34874	not_gl.sig_4b_dn	protein_co	SH3-domain GRB2-like 3
Mcm5	0.062889	-0.63228	not_gl.sig_4b_dn	protein_co	minichromosome maintenance co
Stc2	0.062889	-2.81869	not_gl.sig_4b_dn	protein_co	stanniocalcin 2
Plxnd1	0.063433	-1.11337	not_gl.sig_4b_dn	protein_co	plexin D1
Snu13	0.063621	-0.77285	not_gl.sig_4b_dn	protein_co	SNU13 homolog, small nuclear ribo
Dnajc7	0.064415	-0.61239	not_gl.sig_4b_dn	protein_co	DnaJ heat shock protein family (Hs
Cks1b	0.064415	-0.52685	not_gl.sig_4b_dn	protein_co	CDC28 protein kinase 1b
Rbis	0.064415	-0.59675	not_gl.sig_4b_dn	protein_co	ribosomal biogenesis factor
Satb1	0.064524	-0.92199	not_gl.sig_4b_dn	protein_co	special AT-rich sequence binding p
Tle6	0.064524	-2.11659	not_gl.sig_4b_dn	protein_co	transducin-like enhancer of split 6
BC055324	0.064524	-0.52953	not_gl.sig_4b_dn	protein_co	cDNA sequence BC055324
Cdca7	0.064524	-0.81163	not_gl.sig_4b_dn	protein_co	cell division cycle associated 7
Syt17	0.06453	-2.42802	not_gl.sig_4b_dn	protein_co	synaptotagmin XVII
Dohh	0.06453	-0.57399	not_gl.sig_4b_dn	protein_co	deoxyhypusine hydroxylase/mono
A630031M	0.064538	-2.86779	not_gl.sig_4b_dn	lncRNA	RIKEN cDNA A630031M04 gene
Necab3	0.064809	-1.66591	not_gl.sig_4b_dn	protein_co	N-terminal EF-hand calcium bindin
Wiz	0.06558	-0.82266	not_gl.sig_4b_dn	protein_co	widely-interspaced zinc finger mot
Gm25663	0.06558	-2.01501	not_gl.sig_4b_dn	snoRNA	predicted gene, 25663
Mien1	0.065847	-0.57033	not_gl.sig_4b_dn	protein_co	migration and invasion enhancer 1
Traip	0.065847	-0.71647	not_gl.sig_4b_dn	protein_co	TRAF-interacting protein
Rpl11	0.065847	-0.68496	not_gl.sig_4b_dn	protein_co	ribosomal protein L11

mt-Te	0.065847	-0.75436	not_gl.sig_4b_dn	Mt_tRNA	mitochondrially encoded tRNA glu
Gm49756	0.065847	-1.43669	not_gl.sig_4b_dn	lncRNA	predicted gene, 49756
Poc1a	0.066067	-0.63528	not_gl.sig_4b_dn	protein_co	POC1 centriolar protein A
Adam12	0.066677	-3.70773	not_gl.sig_4b_dn	protein_co	a disintegrin and metallopeptidase
Fbxw7	0.067055	-0.72165	not_gl.sig_4b_dn	protein_co	F-box and WD-40 domain protein
Rnaseh2a	0.067408	-0.59859	not_gl.sig_4b_dn	protein_co	ribonuclease H2, large subunit
Gm49739	0.070059	-0.75461	not_gl.sig_4b_dn	processed_	predicted gene, 49739
H2ac20	0.070605	-0.92832	not_gl.sig_4b_dn	protein_co	H2A clustered histone 20
Gm28911	0.070627	-1.23555	not_gl.sig_4b_dn	processed_	predicted gene 28911
Cdc45	0.070809	-0.69083	not_gl.sig_4b_dn	protein_co	cell division cycle 45
Bax	0.070809	-0.73154	not_gl.sig_4b_dn	protein_co	BCL2-associated X protein
Ddx39a	0.070809	-0.51294	not_gl.sig_4b_dn	protein_co	DEAD box helicase 39a
BC065403	0.071507	-3.65476	not_gl.sig_4b_dn	lncRNA	cDNA sequence BC065403
Lyar	0.071567	-0.68462	not_gl.sig_4b_dn	protein_co	Ly1 antibody reactive clone
Snrpf	0.071911	-0.61835	not_gl.sig_4b_dn	protein_co	small nuclear ribonucleoprotein po
Nr3c1	0.072333	-0.71218	not_gl.sig_4b_dn	protein_co	nuclear receptor subfamily 3, grou
Chaf1b	0.073683	-0.76539	not_gl.sig_4b_dn	protein_co	chromatin assembly factor 1, subu
Ighg2b	0.074569	-2.66097	not_gl.sig_4b_dn	IG_C_gene	immunoglobulin heavy constant ga
1190007I0	0.074577	-0.64258	not_gl.sig_4b_dn	protein_co	RIKEN cDNA 1190007I07 gene
Ikzf3	0.074703	-0.94615	not_gl.sig_4b_dn	protein_co	IKAROS family zinc finger 3
Aunip	0.075172	-1.27762	not_gl.sig_4b_dn	protein_co	aurora kinase A and ninein interac
St3gal6	0.075832	-1.41797	not_gl.sig_4b_dn	protein_co	ST3 beta-galactoside alpha-2,3-sia
Fbl	0.077625	-0.66484	not_gl.sig_4b_dn	protein_co	fibrillarin
Nkrf	0.078447	-0.78072	not_gl.sig_4b_dn	protein_co	NF-kappaB repressing factor
Rbbp7	0.07871	-0.60073	not_gl.sig_4b_dn	protein_co	retinoblastoma binding protein 7,
Sgk3	0.0802	-1.42433	not_gl.sig_4b_dn	protein_co	serum/glucocorticoid regulated ki
Rpl28	0.08165	-0.63873	not_gl.sig_4b_dn	protein_co	ribosomal protein L28
Ier5	0.08165	-0.79652	not_gl.sig_4b_dn	protein_co	immediate early response 5
Espn	0.081692	-1.91737	not_gl.sig_4b_dn	protein_co	espin
Mtg2	0.081692	-0.61874	not_gl.sig_4b_dn	protein_co	mitochondrial ribosome associated
Gm7265	0.081692	-1.52055	not_gl.sig_4b_dn	TEC	predicted gene 7265
Gm5165	0.081854	-0.71005	not_gl.sig_4b_dn	lncRNA	predicted gene 5165
H1f3	0.082508	-1.30348	not_gl.sig_4b_dn	protein_co	H1.3 linker histone, cluster membe
Mcrs1	0.082664	-0.54966	not_gl.sig_4b_dn	protein_co	microspherule protein 1
H2ac13	0.082664	-1.09404	not_gl.sig_4b_dn	protein_co	H2A clustered histone 13
Zfp568	0.083383	-1.22016	not_gl.sig_4b_dn	protein_co	zinc finger protein 568
Ndufb9	0.083737	-0.50194	not_gl.sig_4b_dn	protein_co	NADH:ubiquinone oxidoreductase
Srfbp1	0.083753	-0.73675	not_gl.sig_4b_dn	protein_co	serum response factor binding pro
Rnu4atac	0.083753	-1.28795	not_gl.sig_4b_dn	snRNA	RNA, U4atac small nuclear (U12-de
Mrpl22	0.084062	-0.57414	not_gl.sig_4b_dn	protein_co	mitochondrial ribosomal protein L
Atxn10	0.084401	-0.57236	not_gl.sig_4b_dn	protein_co	ataxin 10
Timm17b	0.084401	-0.53483	not_gl.sig_4b_dn	protein_co	translocase of inner mitochondrial
Emc8	0.085404	-0.50133	not_gl.sig_4b_dn	protein_co	ER membrane protein complex sul
Ndufa12	0.086596	-0.61992	not_gl.sig_4b_dn	protein_co	NADH:ubiquinone oxidoreductase
Nme4	0.086743	-1.61785	not_gl.sig_4b_dn	protein_co	NME/NM23 nucleoside diphospha
Tiam2	0.08768	-2.45848	not_gl.sig_4b_dn	protein_co	T cell lymphoma invasion and met
Top2a	0.088289	-0.50459	not_gl.sig_4b_dn	protein_co	topoisomerase (DNA) II alpha

Sub1	0.088507	-0.75112	not_gl.sig_4b_dn	protein_co	SUB1 homolog, transcriptional reg
Ap3s1	0.088583	-0.64381	not_gl.sig_4b_dn	protein_co	adaptor-related protein complex 3
Chd1	0.089206	-0.69443	not_gl.sig_4b_dn	protein_co	chromodomain helicase DNA bind
Rcor2	0.089882	-1.22511	not_gl.sig_4b_dn	protein_co	REST corepressor 2
Gm45623	0.089882	-4.4247	not_gl.sig_4b_dn	protein_co	predicted gene 45623
Zfp422	0.090235	-0.50061	not_gl.sig_4b_dn	protein_co	zinc finger protein 422
Gm25894	0.091205	-1.22815	not_gl.sig_4b_dn	snoRNA	predicted gene, 25894
Gm48362	0.092022	-3.85474	not_gl.sig_4b_dn	lncRNA	predicted gene, 48362
Rps12	0.093116	-0.5028	not_gl.sig_4b_dn	protein_co	ribosomal protein S12
Cenpn	0.094167	-0.99701	not_gl.sig_4b_dn	protein_co	centromere protein N
Elof1	0.094938	-0.51758	not_gl.sig_4b_dn	protein_co	ELF1 homolog, elongation factor 1
Tcf7	0.095025	-0.54947	not_gl.sig_4b_dn	protein_co	transcription factor 7, T cell specifi
Ssrp1	0.095189	-0.6491	not_gl.sig_4b_dn	protein_co	structure specific recognition prot
Utp11	0.095215	-0.50856	not_gl.sig_4b_dn	protein_co	UTP11 small subunit processome c
Noc3l	0.095663	-0.52716	not_gl.sig_4b_dn	protein_co	NOC3 like DNA replication regulat
Gm44510	0.096245	-0.80907	not_gl.sig_4b_dn	TEC	predicted gene 44510
Psip1	0.097083	-0.51241	not_gl.sig_4b_dn	protein_co	PC4 and SFRS1 interacting protein
Gm19514	0.09712	-2.92997	not_gl.sig_4b_dn	TEC	predicted gene, 19514
Tomm22	0.097175	-0.59519	not_gl.sig_4b_dn	protein_co	translocase of outer mitochondria
Eny2	0.097417	-0.59493	not_gl.sig_4b_dn	protein_co	ENY2 transcription and export con
Mrpl1	0.097838	-0.58177	not_gl.sig_4b_dn	protein_co	mitochondrial ribosomal protein L
Dmrtc2	0.097938	-4.65155	not_gl.sig_4b_dn	protein_co	doublesex and mab-3 related trans
St13	0.098331	-0.60704	not_gl.sig_4b_dn	protein_co	suppression of tumorigenicity 13
Cd8b1	0.098683	-1.44834	not_gl.sig_4b_dn	protein_co	CD8 antigen, beta chain 1
Grk6	0.015536	0.485462	not_gl.sig_5a_up	protein_co	G protein-coupled receptor kinase
Zzef1	0.045786	0.437591	not_gl.sig_5a_up	protein_co	zinc finger, ZZ-type with EF hand d
Sppl3	0.050899	0.365318	not_gl.sig_5a_up	protein_co	signal peptide peptidase 3
Csnk1d	0.065459	0.405873	not_gl.sig_5a_up	protein_co	casein kinase 1, delta
Fkbp15	0.071242	0.389269	not_gl.sig_5a_up	protein_co	FK506 binding protein 15
Uggt1	0.073683	0.445259	not_gl.sig_5a_up	protein_co	UDP-glucose glycoprotein glucosyl
Tbc1d20	0.074703	0.403446	not_gl.sig_5a_up	protein_co	TBC1 domain family, member 20
Ccdc88b	0.075593	0.381869	not_gl.sig_5a_up	protein_co	coiled-coil domain containing 88B
Cacfd1	0.078447	0.46176	not_gl.sig_5a_up	protein_co	calcium channel flower domain co
Erbin	0.081041	0.480772	not_gl.sig_5a_up	protein_co	Erbb2 interacting protein
Mink1	0.08165	0.484893	not_gl.sig_5a_up	protein_co	misshapen-like kinase 1 (zebrafish
Amfr	0.085209	0.423419	not_gl.sig_5a_up	protein_co	autocrine motility factor receptor
Taz	0.08595	0.477393	not_gl.sig_5a_up	protein_co	tafazzin
Lnpep	0.091248	0.439062	not_gl.sig_5a_up	protein_co	leucyl/cystinyl aminopeptidase
Yme1l1	0.000767	-0.49161	not_gl.sig_5b_dn	protein_co	YME1-like 1 (S. cerevisiae)
H3f3a	0.00176	-0.48429	not_gl.sig_5b_dn	protein_co	H3.3 histone A
Hadha	0.00444	-0.43615	not_gl.sig_5b_dn	protein_co	hydroxyacyl-CoA dehydrogenase t
Cetn3	0.004881	-0.46258	not_gl.sig_5b_dn	protein_co	centrin 3
Cfdp1	0.005629	-0.36678	not_gl.sig_5b_dn	protein_co	craniofacial development protein
Sf3b2	0.007149	-0.3257	not_gl.sig_5b_dn	protein_co	splicing factor 3b, subunit 2
Cox7b	0.009834	-0.44826	not_gl.sig_5b_dn	protein_co	cytochrome c oxidase subunit 7B
Aifm1	0.01143	-0.45426	not_gl.sig_5b_dn	protein_co	apoptosis-inducing factor, mitoch
Lsm6	0.011622	-0.45784	not_gl.sig_5b_dn	protein_co	LSM6 homolog, U6 small nuclear

Swi5	0.015536	-0.40723	not_gl.sig_5b_dn	protein_co	SWI5 recombination repair homolo
Mrpl51	0.015888	-0.46299	not_gl.sig_5b_dn	protein_co	mitochondrial ribosomal protein L
Nedd8	0.016456	-0.42845	not_gl.sig_5b_dn	protein_co	neural precursor cell expressed, de
Nfyc	0.016456	-0.44566	not_gl.sig_5b_dn	protein_co	nuclear transcription factor-Y gam
Ssu72	0.017925	-0.39255	not_gl.sig_5b_dn	protein_co	Ssu72 RNA polymerase II CTD phos
Romo1	0.017925	-0.42627	not_gl.sig_5b_dn	protein_co	reactive oxygen species modulator
Ssb	0.020911	-0.3778	not_gl.sig_5b_dn	protein_co	Sjogren syndrome antigen B
Sfr1	0.021138	-0.47972	not_gl.sig_5b_dn	protein_co	SWI5 dependent recombination re
Ctcf	0.025342	-0.41092	not_gl.sig_5b_dn	protein_co	CCCTC-binding factor
Hnrnpk	0.02554	-0.30247	not_gl.sig_5b_dn	protein_co	heterogeneous nuclear ribonucleo
Tnpo3	0.026212	-0.39385	not_gl.sig_5b_dn	protein_co	transportin 3
Rbm8a	0.026659	-0.44615	not_gl.sig_5b_dn	protein_co	RNA binding motif protein 8a
Nsmce4a	0.02728	-0.37476	not_gl.sig_5b_dn	protein_co	NSE4 homolog A, SMC5-SMC6 con
Magoh	0.028741	-0.49544	not_gl.sig_5b_dn	protein_co	mago homolog, exon junction com
Ndufb2	0.028913	-0.44659	not_gl.sig_5b_dn	protein_co	NADH:ubiquinone oxidoreductase
Xpo1	0.030104	-0.49644	not_gl.sig_5b_dn	protein_co	exportin 1
Dnajc8	0.034687	-0.31075	not_gl.sig_5b_dn	protein_co	DnaJ heat shock protein family (Hs
Nol7	0.03672	-0.41133	not_gl.sig_5b_dn	protein_co	nucleolar protein 7
Phax	0.037231	-0.34795	not_gl.sig_5b_dn	protein_co	phosphorylated adaptor for RNA e
Hnrnpl	0.037231	-0.49022	not_gl.sig_5b_dn	protein_co	heterogeneous nuclear ribonucleo
Sgo2a	0.038983	-0.47971	not_gl.sig_5b_dn	protein_co	shugoshin 2A
Polr2b	0.040534	-0.47484	not_gl.sig_5b_dn	protein_co	polymerase (RNA) II (DNA directec
Ndufs5	0.041547	-0.46192	not_gl.sig_5b_dn	protein_co	NADH:ubiquinone oxidoreductase
Cox7a2	0.041994	-0.42042	not_gl.sig_5b_dn	protein_co	cytochrome c oxidase subunit 7A2
Pnrc2	0.042821	-0.47664	not_gl.sig_5b_dn	protein_co	proline-rich nuclear receptor coac
Mrps9	0.043375	-0.44089	not_gl.sig_5b_dn	protein_co	mitochondrial ribosomal protein S
Frg1	0.043715	-0.42526	not_gl.sig_5b_dn	protein_co	FSHD region gene 1
Ppp1r7	0.04653	-0.42288	not_gl.sig_5b_dn	protein_co	protein phosphatase 1, regulatory
Rpl36a	0.047136	-0.44308	not_gl.sig_5b_dn	protein_co	ribosomal protein L36A
Orc2	0.047259	-0.49728	not_gl.sig_5b_dn	protein_co	origin recognition complex, subuni
Mrps14	0.049761	-0.43705	not_gl.sig_5b_dn	protein_co	mitochondrial ribosomal protein S
Mrpl18	0.050342	-0.47307	not_gl.sig_5b_dn	protein_co	mitochondrial ribosomal protein L
Cenpe	0.05143	-0.48773	not_gl.sig_5b_dn	protein_co	centromere protein E
Sf3b5	0.05143	-0.39027	not_gl.sig_5b_dn	protein_co	splicing factor 3b, subunit 5
Smc1a	0.055469	-0.4342	not_gl.sig_5b_dn	protein_co	structural maintenance of chromo
Rpl22	0.058253	-0.37934	not_gl.sig_5b_dn	protein_co	ribosomal protein L22
Ndufa11	0.058629	-0.35342	not_gl.sig_5b_dn	protein_co	NADH:ubiquinone oxidoreductase
AC159819.	0.058629	-0.45551	not_gl.sig_5b_dn	protein_co	ubiquitin-conjugating enzyme E2S
Hdac1	0.059679	-0.47908	not_gl.sig_5b_dn	protein_co	histone deacetylase 1
Bud31	0.061229	-0.3749	not_gl.sig_5b_dn	protein_co	BUD31 homolog
Cdc37	0.062572	-0.36701	not_gl.sig_5b_dn	protein_co	cell division cycle 37
Ndufa7	0.062889	-0.35844	not_gl.sig_5b_dn	protein_co	NADH:ubiquinone oxidoreductase
Zfp26	0.064415	-0.39666	not_gl.sig_5b_dn	protein_co	zinc finger protein 26
Ndufs8	0.064524	-0.4944	not_gl.sig_5b_dn	protein_co	NADH:ubiquinone oxidoreductase
Snw1	0.06579	-0.37067	not_gl.sig_5b_dn	protein_co	SNW domain containing 1
Rps29	0.06579	-0.47107	not_gl.sig_5b_dn	protein_co	ribosomal protein S29
H2ac11	0.065847	-0.45533	not_gl.sig_5b_dn	protein_co	H2A clustered histone 11

Pcnp	0.066829	-0.40225	not_gl.sig_5b_dn	protein_co	PEST proteolytic signal containing
Ndufb5	0.067055	-0.3555	not_gl.sig_5b_dn	protein_co	NADH:ubiquinone oxidoreductase
Cbx3	0.067408	-0.37394	not_gl.sig_5b_dn	protein_co	chromobox 3
U2surp	0.069158	-0.2573	not_gl.sig_5b_dn	protein_co	U2 snRNP-associated SURP domai
Rars	0.069446	-0.44109	not_gl.sig_5b_dn	protein_co	arginyl-tRNA synthetase
Bms1	0.072538	-0.37205	not_gl.sig_5b_dn	protein_co	BMS1, ribosome biogenesis factor
Snrpc	0.074577	-0.38544	not_gl.sig_5b_dn	protein_co	U1 small nuclear ribonucleoprotei
Commd1	0.075172	-0.37824	not_gl.sig_5b_dn	protein_co	COMM domain containing 1
Rsrc2	0.076213	-0.33436	not_gl.sig_5b_dn	protein_co	arginine/serine-rich coiled-coil 2
Kif22	0.076883	-0.4932	not_gl.sig_5b_dn	protein_co	kinesin family member 22
Kat7	0.078447	-0.38573	not_gl.sig_5b_dn	protein_co	K(lysine) acetyltransferase 7
Mettl23	0.079492	-0.34339	not_gl.sig_5b_dn	protein_co	methyltransferase like 23
Uqcr10	0.080512	-0.41064	not_gl.sig_5b_dn	protein_co	ubiquinol-cytochrome c reductase
Prpf40a	0.08595	-0.27351	not_gl.sig_5b_dn	protein_co	pre-mRNA processing factor 40A
Bod1l	0.08613	-0.29014	not_gl.sig_5b_dn	protein_co	biorientation of chromosomes in c
Hnrnpul2	0.08613	-0.38647	not_gl.sig_5b_dn	protein_co	heterogeneous nuclear ribonucleo
Smarca5	0.086391	-0.38625	not_gl.sig_5b_dn	protein_co	SWI/SNF related, matrix associate
Mki67	0.086801	-0.42583	not_gl.sig_5b_dn	protein_co	antigen identified by monoclonal a
Cdca8	0.088025	-0.46461	not_gl.sig_5b_dn	protein_co	cell division cycle associated 8
Rpl24	0.090594	-0.42104	not_gl.sig_5b_dn	protein_co	ribosomal protein L24
Rpa1	0.091248	-0.46161	not_gl.sig_5b_dn	protein_co	replication protein A1
Kars	0.092498	-0.37672	not_gl.sig_5b_dn	protein_co	lysyl-tRNA synthetase
Pop4	0.09316	-0.45837	not_gl.sig_5b_dn	protein_co	processing of precursor 4, ribonuc
Selenoh	0.093219	-0.47735	not_gl.sig_5b_dn	protein_co	selenoprotein H
Rpl7l1	0.098588	-0.35861	not_gl.sig_5b_dn	protein_co	ribosomal protein L7-like 1
Rpl6	0.09887	-0.30357	not_gl.sig_5b_dn	protein_co	ribosomal protein L6

d, actin dependent regulator of chromatin, subfamily d, member 2

d, actin dependent regulator of chromatin, subfamily a, member 5
