# Supplementary material for: Apobec-mediated retroviral hypermutation in vivo is dependent on mouse strain
Source: PLoS Pathog. 2024 Aug 29;20(8):e1012505. doi: 10.1371/journal.ppat.1012505 (PMC11389910; doi:10.1371/journal.ppat.1012505)
Supplement: Attachment — Submitted filename: Byun_TBLV_B6_Rebuttal_v3.pdf [file ppat.1012505.s004.pdf]

MOLECULAR BIOSCIENCES
College of Natural Sciences

100 E 24th Street, NHB 2.606 • A5000 • Austin, Texas 78712 • 512-471-5105 • FAX 512-471-1218
molecularbiosci.utexas.edu

March 24, 2024

Re: Revision of PPATHOGENS-D-23-02011

Dear Dr. Ross,

Thank you for the opportunity to submit a revised version of our manuscript "Apobec-Mediated Retroviral Hypermutation In Vivo is Dependent on Mouse Strain" to *PLoS Pathogens*. The summary comments indicate that reviewers #1 and 2 want additional mechanistic insights that explain differences between proviral mutagenesis after infection of BALB/c compared to C57BL/6 (B6) mice. Although we have added some additional experiments, we believe that the explanation is likely a complex one and is beyond the scope of this paper. Comments from each reviewer have been addressed below.

Reviewer #1:

Summary/Major issues: This reviewer states that our paper is well-written and that conclusions are largely justified. However, additional mechanistic details have been requested. (1) Reviewer #1 states that we should purify virus and measure viral RNA in the tumors rather than measuring proviral DNA. In general, viral RNA levels are proportional to viral DNA levels, which have been measured here. (2) We did not observe large differences in size and weight of tumors. This information has been added to Materials and Methods. (3) The statement in lines 148-149 has been eliminated. (4) Figures 2D-F have measured levels of viral DNA relative to the levels of endogenous *Mtv*s. Endogenous *Mtv*s are diploid relative to haploid TBLV integrations. The results of certain infections were not detectable above endogenous viral DNA levels. This statement has now been added to the figure legend. (5) A good infectivity assay in cell culture is not available for TBLV or MMTV. Serial dilutions to determine virus infectivity from each tumor is not feasible *in vivo*. (6) Examination of the previous Sanger sequencing data of ~2 kb proviral clones has not revealed any specific hotspots in proviruses obtained from either BALB/c or B6 mice. (7) Regarding the analysis of additional knockout strains, we lost the mouse Apobec3 (mA3)-knockout strain on the B6 background during the COVID outbreak. No significant differences were seen between proviral mutations in the presence or absence of Rem expression or by comparisons of proviruses derived from wild-type B6 or AID-knockout mice on the B6 background. However, new data from the analysis of proviruses from B6 mA3/AID double-knockout mice revealed significantly reduced G-to-A mutations with those from either wild-type B6 or AID-knockout mice. These results suggest that mA3 is responsible for many G-to-A mutations regardless of Rem expression. (8) The reviewer suggests that the observed mutations may be due to reverse transcriptase (RT). Although MMTV/TBLV RT has not been extensively characterized, the same RT enzyme is present in TBLV-WT and TBLV-SD used to infect both B6 and BALB/c mice. Therefore, it doesn't explain why we would see differences in proviral mutations only in BALB/c mice. (9) The results in Fig. 7 have been quantified, and the figure has been modified. The figure legend has been updated to state that this experiment has been repeated three times. (10) We have infected BALB/c and B6 splenocytes *ex vivo* with TBLV. However, infection levels were very low and undetectable by PCR, similar to the results observed early after *in vivo* infection. Note that MMTV and TBLV infection does not cause viremia. (11) Lines 341-344 – The RNA analysis in B6 tumors was designed to determine if there was a difference in *Apobec* transcript levels in the presence and absence of Rem. Instead, the results showed differences in cytokine/growth factor mRNAs, but not *Apobec* transcripts. We have not performed this analysis for TBLV-infected BALB/c tumors since it does not address the primary focus of this study. (12) Both *Stat1* and *Stat4* mRNAs were statistically increased (Fig. 8), although *Stat4* mRNA levels showed the highest relative increase between TBLV-WT and TBLV-SD tumors. (13) Lines 381-382 - We addressed the difference between B6 and BALB/c mice and its effect on tumorigenesis in the second paragraph of the Discussion.

(14) Lines 388-389 - Characterization of the integration patterns after TBLV-WT and SD infection is beyond the scope of this manuscript. However, we did not see dramatic alterations in the numbers of LTR enhancer repeats in tumors induced by wild-type and Rem-null TBLV in wild-type B6 and AID-knockout mice. We previously have shown that these repeats drive proto-oncogene expression (Broussard et al., *JV*, 2002). (15) Lines 395-396 have been rewritten as requested. (16) Lines 406-407 - We have speculated that mA3 levels are higher in immature B cells due to increased proviral loads in µMT tumors since µMT mice lack mu immunoglobulin heavy chain and conventional B-2 cells. However, characterization of immature B cells in BALB/c and B6 mice is beyond the scope of this study.

Minor issues: (1) As requested, we have expanded the description of the µMT mice in the Abstract, Results, and Materials and Methods. (2) Line 181 – This sentence was speculation about the observed results and has been moved to the Discussion. (3) Lines 223-225 – This sentence has been revised. (4) The data in Fig. 3 are from 3 different tumors for each viral clone and mouse strain tested (between 50-55 independent clones for each infected strain). This information has been added to the figure legend. (5) Line 259 – The value has been corrected to $p<0.0001$. (6) Lines 284 and 287 – The MiSeq data are from high-dose infections, and the Nanopore data are from low-dose infections. The inconsistency has been corrected. (7) Lines 409-410 – As requested, we have provided an explanation for the difference in AT<u>C</u>-motif mutations in proviruses from µMT tumors in the Discussion.

Reviewer #2:
Summary/Major issues: This reviewer states that we have not demonstrated strain-dependent differences in proviral DNA mutations, and that our observations are "preliminary". We strongly disagree with these statements since our data reveal the lack of proviral mutagenesis using cloning and Sanger sequencing as well as high throughput sequencing after infection of B6 mice at two different doses. These results are in stark contrast to our published results in BALB/c mice (Singh et al, *mBio*, 2019). We have added new *ex vivo* experiments to Fig. 7 (also Fig. S1) as well as new high throughput sequencing of proviruses from mA3-AID double-knockout mice (Fig. 5). (1) The reviewer requested a time course for mA3 and AID expression in B6 and BALB/c splenocytes, which we previously had performed with fewer time points, but not provided. The results now are shown in Fig. 7. Only a slight response to ConA is observed for AID levels (Fig. S1). As suggested, mA3 levels are upregulated prior to AID levels. We have not maintained µMT mice in our colony since before the COVID epidemic due to breeding difficulties. As the reviewer notes, multiple experiments may be proposed to follow up on these results, including differences in promoter/enhancer sequences for the *Aicda/mA3* genes in the two strains. (2) The gel in Fig. 6B has been improved. All TBLV-induced tumors are polyclonal, unlike MMTV-induced mammary tumors (Broussard et al., *JV*, 2002). (3) Tumor-free status was defined by the period when the animals show no symptoms. Unlike mammary tumors, which can be palpated, T-cell lymphomas were detected by their restriction of lung function/breathing of the animals. Mice are sacrificed immediately since they die quickly (12-24 hr) after these symptoms are observed. The survival plots in Fig. 2C are based on inoculation of 20 to 22 animals for each virus/mouse strain combination. This information has been added to the legend and/or Materials and Methods.

Minor issues: (1) Figures 2A-C are plotted from 0, but the line starts slightly internal to the axes. In Fig. 2E, the values have been corrected. Tumor development is the result of more than viral load, although increased viral integrations would be expected to increase insertion near proto-oncogenes. MMTV and TBLV are known to activate different proto-oncogenes by enhancer insertion. These two viruses have different enhancers (Fig. 1), and the target cell may influence insertion sites. (2) Line 148, the sentence has been eliminated. As mentioned above, line 181 has been moved to the Discussion. Lines 230-231 – the phrase "*Aicda*-insufficient" has been changed to "*Aidca*-deficient". Lines 249-250 – Previous wording stated that the results "suggest" that mutations occur in immature B cells, which are increased in µMT mice. This sentence also has been moved to the Discussion. Line 258 – The sentence has been reworded to indicate that tumors induced by TBLV-SD occurred more rapidly than those induced by TBLV-WT at the lower viral dose. Line 312 – Type I interferons often are produced after viral infection. The phrase "simulate a viral infection" has been removed. (3) "Activation-stimulated" was not found in the manuscript. Line 317 – The phrase containing "slighter greater" has been eliminated in the revised manuscript.

Reviewer #3:
Summary/Major issues: (Comments 1 and 2) The reviewer has requested a table summarizing differences between B6 and BALB/c mice including proviral mutations in the absence of Rem and known characteristics of mA3 and AID in the two strains. No isoform differences have been noted between the *Aicda* genes from BALB/c and B6 mice. Table 4 has been provided to summarize characteristics known from our experiments and those previously published. The reference to enhanced mA3 levels in B6 relative to BALB/c mice has been added. No new experiments were requested.

Minor issues: (3) Lines 223, 247 – Previous publications have suggested that SYC and ATC-motif mutations are associated with AID and mA3, respectively. However, we previously noted that these mutations are affected by Rem differently than proviral mutations primarily associated with AID and mA3 (WRC and TYC-motifs, respectively) from TBLV-infected BALB/c tumors (Singh et al., *mBio*, 2019). Examination of the Sanger sequencing data from high-dose infections of B6 mice also showed that knockout of AID did not have the same effect on WRC and SYC-motif proviral mutations in the absence of Rem expression. Similarly, the TYC and SYC-motif proviral mutations were not affected in the same manner in the presence and absence of Rem. For example, in wild-type B6 tumors, the TYC-motif mutations were not significantly different, whereas the ATC-motif mutations were significantly different. The opposite was observed in µMT mice. If mA3 was responsible for mutations in both ATC and TYC-motifs, the absence of Rem should have affected both types of mutations in the same way. This has been clarified in the revised manuscript. (4) We have added that G-to-A and C-to-T mutations are typical of Apobecs in the Abstract. The µMT mice have been described in the revised text. (5) Fig.1B – The positions of the primers used for amplification of the LTR repeats as well as the *env*-LTR fragment have been added. The figure legend has been modified to explain the enhancer triplication in the TBLV LTR and the "X" mutation in TBLV-SD. Sequences flanking a deleted region of negative regulatory elements in the MMTV LTR are triplicated in the TBLV LTR. (6) Fig. 8C and line 359 – The text has been modified to correspond with the chart, which cites "inflammation mediated by chemokine and cytokine signaling". (7) Line 207 – The WRC motif was defined as "AID-associated" earlier (line 90) in the original text.

In summary, we are grateful to the reviewers for suggestions to improve our manuscript. We have provided additional data and addressed all reviewer comments. Both reviewers #1 and 3 indicated that our conclusions were justified. Some requested experiments are beyond the scope of this study. Overall, our manuscript elucidates an important issue affecting studies of mouse strain-specific innate immune responses to many pathogens.

Sincerely,

Jaquelin P. Dudley
Professor of Molecular Biosciences
Associate Director,
LaMontagne Center for Infectious Disease
512-471-8415 (Office phone)
jdudley@austin.utexas.edu (E-mail)
