# Supplementary material for: Apobec-mediated retroviral hypermutation in vivo is dependent on mouse strain
Source: PLoS Pathog. 2024 Aug 29;20(8):e1012505. doi: 10.1371/journal.ppat.1012505 (PMC11389910; doi:10.1371/journal.ppat.1012505)
Supplement: Attachment — Submitted filename: Byun_TBLV_B6_Rebuttal_6-24v2.pdf [file ppat.1012505.s005.pdf]

MOLECULAR BIOSCIENCES
College of Natural Sciences

100 E 24th Street, NHB 2.606 • A5000 • Austin, Texas 78712 • 512-471-5105 • FAX 512-471-1218
molecularbiosci.utexas.edu

August 1, 2024

Re: PPATHOGENS-D-23-02011 – Revision 2

Dear Dr. Ross,

Thank you for the opportunity to submit a further revision of our manuscript "Apobec-Mediated Retroviral Hypermutation In Vivo is Dependent on Mouse Strain" to *PLoS Pathogens*. Reviewer #1 indicates that examination of deaminase protein levels in tumors is necessary to strengthen our results, whereas Reviewer #2 requests more careful interpretation of our results. We have provided additional data on Apobec3 levels in tumors and sequencing of PCR products from isolated virion RNA and have rewritten parts of the text as indicated below.

Reviewer #1:
Summary/Major issues: (1) This reviewer requested examination of mutations within virion RNAs purified from tumors. Virions were purified from frozen tumor samples, and viral RNA was extracted. The yields of virion RNA after DNase treatment were similar for all tumors. RNA was used for reverse transcription-PCR with primers for the same ~2 kb envelope-LTR region analyzed for proviral DNA. The resulting PCR product was purified and used for Nanopore sequencing and analysis. Although the numbers of reads obtained were much lower from the products of virion RNA as compared to products from proviral DNA, the results showed that G-to-A changes were dominant in both types of samples. However, the mutational changes observed in virion RNA were quite variable between tumors, precluding statistical analysis between groups (see new Fig. 5C). (2) As suggested, we also extracted protein from representative tumor samples from TBLV-WT and TBLV-SD-induced tumors for analysis by Western blotting. We could detect mA3 at approximately equal levels in tumors induced in B6 and B6 AID-knockout tumors regardless of whether TBLV expressed Rem (new Fig. S1). AID protein expression was not detected in any of these T-cell lymphomas. (3) The comment in the Results section about mA3 levels in immature B cells was speculative and has been removed. The µMT mice were used to show that MMTV and TBLV differ with respect to their requirement for infection of mature B cells.

Minor issues: None listed.

Reviewer #2:
Summary/Major issues: (1) The focus of this paper is on the differences between Apobec-mediated proviral mutations in B6 and BALB/c mice after infection with retroviruses that differ with respect to Rem expression. The purpose of µMT mouse infections was to determine whether TBLV has a requirement for mature cells during *in vivo* replication. Unlike MMTV, TBLV lacks this requirement. We agree with the reviewer that mA3 is the obvious mutagen, which we previously stated in the Discussion. We have added a statement about the high levels of mA3 in B6 mice, which is also given in Table 4. (2) As the reviewer notes, experiments with splenocytes show variability of AID and mA3 expression even using pools of cells from multiple animals. The references cited by the reviewer show only B6 AID mRNA levels (Kuraoka et al., *Cell Reports*, 2015) or protein levels in an engineered B6 strain (Pone et al, *JI*, 2015). In the latter study, the numbers of repetitions or numbers of individual mice tested for AID protein levels were not described. We have omitted the supplementary figure about AID levels and have included a scatterplot summarizing multiple experiments and the variation observed even using pooled splenocytes in each experiment. In addition, we have provided a new Fig. 7 with all samples analyzed on the same gel. (3) We have removed the term "immature B cells", except for that cited for human AID expression (T. Cantaert et al., *Immunity*,

2015). The phenotype of B cells detected in μMT mice (M.K. Lagerquist et al., *JBMR Plus*, 2022) has been described in the Discussion. (4) The indicated references have been modified as requested.

Minor issues: (a) Line 36 has been corrected. (b) The repetition in line 39 has been corrected. (c) Lines 43-44 have been reworded. (d) Line 90 – "associated" has been corrected to "activated". (e) Lines 106-107 – The sentence has been clarified. (f) The sentence has been reworded. (g) Line 142 – corrected. (h) Lines 153-154 – corrected. (i) Line 160 – corrected. (j) TBLV-SD has been used in most instances in the revised manuscript. (k) Line 236 – corrected. (l) Line 268 – "tumor development" has been substituted in this sentence. (m) Line 273 – The wording has been modified as suggested. (n) Line 274 – corrected. (o) Lines 280-281 – Although we don't know the origin of these mutations, it is likely to be a cytidine deaminase. We have inserted "cytidine deaminase", instead of "enzyme". (p) Line 332 – The sentence has been reworded for clarity. We are comparing uncultured splenocytes to splenocytes cultured in the presence of IL-4/LPS.

In summary, we appreciate suggestions to improve our manuscript. We have provided additional data and addressed reviewer comments. Some requested experiments are beyond the scope of this study, which now has 8 figures with multiple panels and 4 tables plus supplementary data. Our manuscript elucidates how innate immune responses to the same viral infection vary in different mouse strains and highlights a unique function of AID that is manipulated by MMTV-encoded Rem.

Sincerely,

Jaquelin P. Dudley

Jaquelin P. Dudley
Professor of Molecular Biosciences
Associate Director,
LaMontagne Center for Infectious Disease
512-471-8415 (Office phone)
jdudley@austin.utexas.edu (E-mail)
